# Route effects in city-based survey knowledge estimates

**DOI:** 10.1007/s10339-022-01122-0

**Published:** 2023-01-23

**Authors:** Jakub Krukar, Samuel Navas Medrano, Angela Schwering

**Affiliations:** Institute for Geoinformatics, University of Muenster, Heisenbergstr. 2, 48149 Muenster, Germany

**Keywords:** Direction estimation, Survey knowledge, Urban environments, Pointing task, Sketch maps

## Abstract

When studying wayfinding in urban environments, researchers are often interested in obtaining measures of participants’ survey knowledge, i.e., their estimate of distant locations relative to other places. Previous work showed that distance estimations are consistently biased when no direct route is available to the queried target or when participants follow a detour. Here we investigated whether a corresponding bias is manifested in two other popular measures of survey knowledge: a pointing task and a sketchmapping task. The aim of this study was to investigate whether there is a systematic bias in pointing/sketchmapping performance associated with the preferred route choice in an applied urban setting. The results were mixed. We found moderate evidence for the presence of a systematic bias, but only for a subset of urban locations. When two plausible routes to the target were available, survey knowledge estimates were significantly biased in the direction of the route chosen by the participant. When only one plausible route was available, we did not find a statistically significant pattern. The results may have methodological implications for spatial cognition studies in applied urban settings that might be obtaining systematically biased survey knowledge estimates at some urban locations. Researchers should be aware that the choice of urban locations from which pointing and sketchmapping are performed might systematically distort the results, in particular when two plausible but diverging routes to the target are visible from the location.

**Supplementary Information:**

The online version contains supplementary material available at 10.1007/s10339-022-01122-0.

Spatial knowledge of large environments is understood to consist of survey knowledge, route knowledge, and landmark knowledge (Ishikawa and Montello 2006; Kim and Bock 2021; Montello 1998; Siegel and White 1975). Survey knowledge is configurational knowledge about locations within distant parts of the environment. Although often referred to as a “cognitive map”, it is distorted and does not resemble a topographic map. In order to reflect this, Tversky proposed to use the term “cognitive collage” instead (Tversky 1993). Many distortions in human cognitive collages follow consistent, repeatable patterns McNamara et al. (1989). Understanding these consistent distortions is a valuable line of research for theoretical and applied reasons: it contributes to our understanding of human knowledge structure, and it may help to design environments, technologies, and educational practices that allow people to form more accurate mental representations of their environments.

One commonly recognised consequence of imperfect cognitive collages are distorted direction estimations to distant targets. Researchers have studied factors that cause consistent distortions in direction estimations, in particular the influence of the environmental structure (Freundschuh 1992). One example is the hierarchical organisation of space, i.e., the fact that places can belong to higher-level conceptual or administrative categories (e.g. cities belong to regions and countries). Stevens and Coupe (1978) showed that the spatial relation between the superordinate categories causes a bias in direction estimates between individual places: participants use the higher-level spatial relation to judge individual distances. However, experiments based on naturalistic urban environments are challenged by the fact that not all sources of potential distortions are obvious, known, or quantifiable. For example, conceptual higher-level categories might have vague boundaries or be different for different participants (Montello et al. 2003).

Schwering et al. (2017) described a dataset that shows signs of a systematic bias in city-based pointing estimates, but the source of this bias is speculative. In their paper, participants were divided into two groups: the control group used a classical pedestrian navigation system; the experimental group used a system that additionally visualised the direction to distant landmarks in the city on the edge of its screen. At the end of the navigational walking task, participants were asked to point to distant well-known landmarks in the city. It was expected that pointing to distant landmarks would have wider distribution (larger error) among the control group that did not see the additional off-screen visualisation. Surprisingly, in the case of two city landmarks, pointings of the control group had a bimodal distribution instead of a wider unimodal one (Fig. 1)—a sign of systematic errors (Meilinger et al. 2016). The authors suggested the following interpretation: both landmarks that resulted in such bimodal distributions are located across a large barrier (a lake and a rail road) that would force the participants to take a significant detour should they have to walk there. Barriers dissociate the direction of the route with the correct direction of the pointing and could result in a consistent pointing error biased towards the direction of the route. Note that such an effect could manifest itself regardless of the route knowledge held by the participant because initial segments of the routes are visible from the location of the pointing and do not need to be retrieved from memory.

Methodologically, this would present a challenge to applied spatial cognition studies in urban environments. Participants of such studies explore the environment and perform direction estimation tasks as a measure of their spatial knowledge about that space. Researchers do not routinely control for the potential effect of barriers located between the participant and the target of those estimations. If such an effect was found, it would imply that some testing locations may be consistently associated with less accurate responses.

Barriers to movement have already been investigated as a source of spatial distortions, although primarily in the context of distorted distance estimates. One explanation of their effect is the hierarchical organisation of space (Stevens and Coupe (1978)) because being located before vs behind a barrier groups landmarks into separate categories (Hirtle and Jonides 1985). However, this explanation could account for different distortions towards targets before vs behind a barrier but not for the pattern of leftwards- vs rightwards- bias for targets located across a barrier (as is the case in Fig. 1). Thus, an alternative explanation is needed. One plausible possibility is the “route effect”, i.e., the fact that the memory of spatial relations between two locations can be distorted by the shape and distance of the route connecting them.

“Route effects” have been shown for distance (but not direction) estimates. For instance, McNamara et al. (1984) showed that route distance between two cities (i.e., the cumulative length of roads connecting the cities) has a larger impact on subjective distance estimates between those cities than the Euclidean distance (i.e., “as the crow flies”). Lederman et al. (1987) showed that the distance walked along a path (but not the time spent on it) is associated with errors in Euclidean distance estimates between targets on the path. Klippel et al. (2005) further developed this argument demonstrating that the “route effect” generalises to more abstract perceptual tasks that simply emphasise the connectedness of abstract symbols and do not even contain actual “routes”.

The fact that the “route effect” occurs in distance estimates does not guarantee that it will manifest itself in direction estimates. These two measures asses distinct aspects of the underlying mental representation of space. First, they are typically performed in different reference frames: judging distances between places in the allocentric reference frame, while pointing performance requires using the egocentric reference frame (Klatzky 1998). People differ with respect to their abilities of and preferences for using these distinct strategies (Münzer et al. 2016). Second, even when the disparity of reference frames is eliminated, the measures give distinct results. Distance estimates can be performed from the egocentric perspective if the participant is asked to judge how far a target is from the place they are currently standing at. The work of Hegarty et al. (2006) that employed such a method showed a correlation of *r* = .67 between distance and direction estimates. This is a relatively low result, given the fact that participants made distance and direction estimates one after another, from the same place in the environment, to the same set of targets.

Thus, the presence of “route effect” within direction estimations seems possible but cannot be assumed based on the results from distance estimation tasks alone. Theoretically, the “route effect” could explain the leftward- vs rightward- biases in direction estimations in urban environments because the presence of barriers dissociates the true direction to the target from the direction of the route that needs to be taken in order to reach it. In the presence of barriers, a person wishing to reach a target across it must plan a detour, significantly veering away from the true direction. Thus, walking to a target located across a barrier inevitably involves an action of walking either far to the left or to the right, around that barrier.

This fact might affect direction estimates if spatial memories preserve perceptuomotor information about actions and experiences in the environment, as has been postulated by many (Brunyè et al. 2012; Sadalla and Montello 1989; Wang et al. 2012, 2020). Such an explanation would be consistent with the embodied (Tversky 2009) and grounded (Barsalou 2008) views on human cognition. Within these views, the properties of the human body (e.g. its necessity to physically circumnavigate barriers) and available actions (e.g. going left vs right) are believed to have an impact on the seemingly internal mental processes, such as estimating the direction to a distant target from memory. Routes available to circumnavigate barriers restrict available actions (Jeffery 2021). Specifically, the direction of the initial segment of the route has been shown to weight particularly heavily on human navigational decision making (Bailenson et al. 2000; Dalton 2003). This paper focuses on the direction of the initial segment of the route as a predictor of systematically biased survey knowledge estimates. We focus exclusively on situations in which two or more distinct route alternatives are clearly visible from the location of pointing. This ensures that the role of participants’ heterogeneous spatial knowledge is reduced in the evaluation of this potentially embodied effect.

The above literature shows that there are theoretically plausible reasons for which barriers might consistently bias direction estimates. Some distortions in survey knowledge caused by barriers and deviating routes have already been shown with distance estimates. To our knowledge, however, the impact of barriers on direction estimates has not been yet studied, except for the accidental finding discussed by Schwering et al. (2017). The presence of barriers in the urban environment provides a natural opportunity to investigate the effect of embodied and grounded property of human cognition on direction estimation.

In this line of research, the choice of method with which participants are asked to externalise their mental representation of space might have a significant impact on the conclusions (Richardson et al. 1999). Direction estimations are most commonly obtained with two methods: pointing task and sketchmapping. The pointing task involves participants pointing sequentially in the direction of the estimated targets and it is the gold standard method of survey knowledge estimation (Montello 2016; Montello et al. 1999). Sketchmapping is another commonly used method in spatial cognition research (Krukar et al. 2018; Schwering et al. 2022), where participants are asked to draw a simplified map of the environment. If their own location and the position of distant targets are included in the sketch, researchers can extract angular deviation between the true and the estimated direction to the targets.

Pointing and sketchmapping tasks differ substantially, even though they both require the use of survey knowledge. Pointing task is performed in the egocentric perspective. This means that participants are using their own body orientation, as well as the information visible from their current perspective to estimate pointing directions. Sketchmapping is different because it is performed in the allocentric perspective (in the top-down view), and therefore allows and promotes the use of spatial relations between objects to estimate the location of other objects (Montello 1991).

Because of these different task characteristics, the method used to extract survey knowledge may impact the bias present in the direction estimates (Kitchin 2000; Kitchin and Fotheringham 1997; Montello 2016; Montello et al. 1999). For example, using sketchmapping might reduce the bias possibly occurring across barriers, because it provides an instant, continuous feedback on the sketched location of each target, in comparison with other targets. When sketching the estimates on the map, the participant has a chance to maintain a subjectively acceptable coherence of the set of estimates, searching for the most acceptable overall solution (Montello 1991). Conversely, the pointing task is performed without direct access to other estimates and can be more sensitive to biases that affect some targets but not others.

The current manuscript reports the result of an exploratory experiment designed to systematically study the effect reported by Schwering et al. (2017). We describe an experiment in which participants pointed to numerous targets from different locations across the city of Muenster, Germany. We selected targets that in some cases were separated from the participants’ location by prominent barriers to movement such as water bodies or rail tracks. This experimental design ensured that the route choice available to participants to reach these targets varied in terms of its angular deviation from the true direction. Taking this angular deviation of routes into account, we investigated whether preferred route choice is associated with a systematic bias in pointing/sketchmapping performance.

Testing this is difficult in practical terms because it requires collecting data from multiple locations in a city. This introduces uncontrolled variance across locations and possibly across differently biased groups of participants that would be recruited at each location. In order to tackle this challenge, the current paper uses a virtual reality (VR) set-up that allows participants in the laboratory to experience multiple locations, at distant areas of the city, within the duration of a single experimental session. Virtual reality applications have become commonplace in spatial cognition studies (Kuliga et al. 2015). Survey knowledge data collected in VR have been shown to correlate with survey knowledge data collected in the traditional manner. For pointing, Waller et al. (2004) observed the correlation of *r* = 0.87 between pointing in the real-world setting and inside the VR-based photographic presentation of the same environment. Hegarty et al. (2006) found a relatively low correlation of .41 between direction estimations in a real-world task and direction estimations in a VR-based task; as well as a .33 correlation between sketchmapping performance in a real-world task and a VR-based task. However, in this study the virtual environment was not designed to represent or correspond to the real-world condition. van der Ham et al. (2015) showed higher performance in the real-world environment compared to the VR condition in both pointing and sketchmapping task. The route in the VR condition of this study was similar but differed in the length of some segments, compared to the real-world route. In the experiment by Richardson et al. (1999), the virtual environment was designed to represent the real-world one. Participants who explored the space in VR had higher pointing errors. However, to their disadvantage might have played the fact that testing always took place in the real environment. Thus, results on the correspondence of pointing and sketchmapping tasks in real and virtual environments are mixed. The current study utilises a procedure most similar to this tested by Waller et al. (2004), who obtained a high correlation in pointing task results but has not tested the sketchmapping task. It therefore seems that the validity of virtual environments for the experimental procedure employed in our study requires further confirmation.

**Fig. 1:**
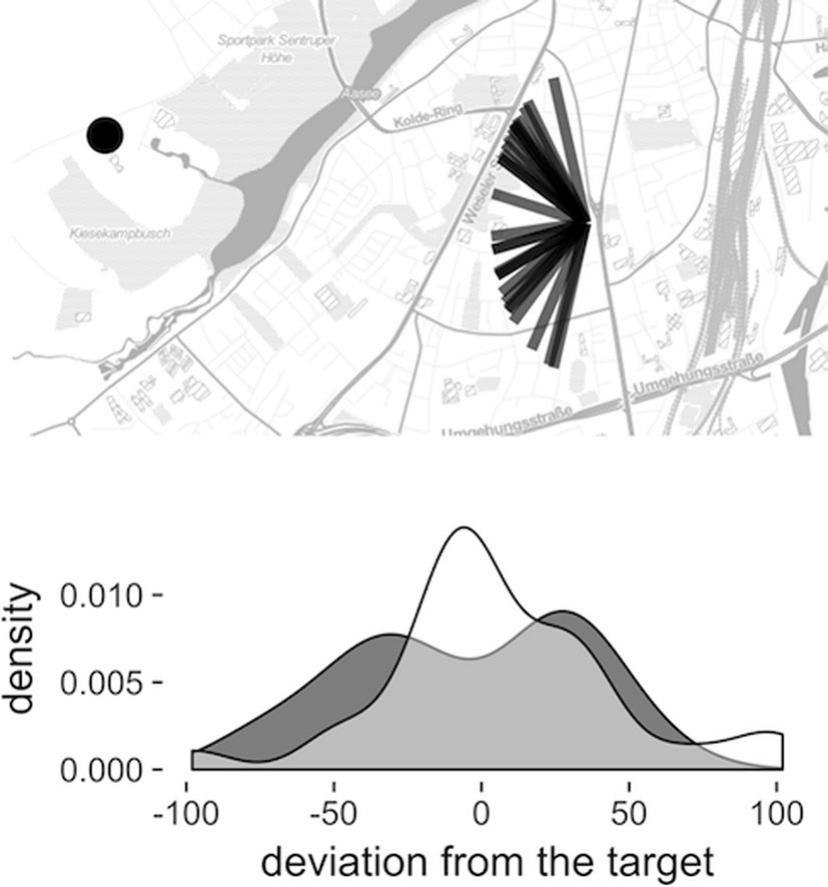
Part of the figure originally presented in Schwering et al. (2017). Top: All pointings to the zoo (indicated with a dot) made by the control group. Bottom: the comparison of the pointing distribution across the control (grey) and experimental (white) group. Note the bimodal distribution of the control group

## Aim of this study

The aim of this study was to investigate whether there is a systematic bias in pointing/sketchmapping performance associated with the preferred route choice in an applied urban setting. Our experimental set-up required a validation of a virtual reality-based data collection method.

We hypothesised that a pointing and sketchmapping task performed in virtual reality (VR) will result in similar pointing- and sketchmap-based estimates, compared to data collected on the streets of the city (Hypothesis 1). In the subsequent, fully VR-based experiment, we hypothesised that pointing to/sketchmapping of distant targets will be biased in the direction of the chosen route (Hypothesis 2).

We present two experiments. Experiment 1 validated the procedure for collecting pointing and sketchmapping data. In it, we tested whether pointing and sketchmapping data collected in our laboratory-based virtual reality setting will follow the same distribution as data collected in the real-world context of the city location. Experiment 2 used the validated procedure to test Hypothesis 2.

## Experiment 1: method validation

In Experiment 1, we validated the VR-based procedure by collecting pointing and sketchmapping data to the same four targets, from a single location, in two between-subject conditions: in *real-world* and in *virtual reality (VR)*. While using VR-specific equipment, we did not present computer graphic simulations typical to VR, but rather high-resolution panoramic photographs of real city locations, similarly to the *immersive video environment* paradigm (Ostkamp and Kray 2014; Singh et al. 2006).

### Methods

#### Participants

Participants in the *real-world* condition were recruited from passers-by at the location of interest in the city of Muenster, Germany. We recruited 37 participants (mean age = 35.49, SD = 16.80), of which 26 were female and 2 did not declare gender. Participants in the *VR* condition were recruited via our institute’s communication channels. We recruited 22 participants (mean age = 32.18, SD = 9.99), of which 8 were female. Participants were not paid in either condition. Sample size determination was opportunistic, restricted by the available time and resources.

#### Material

**Fig. 2:**
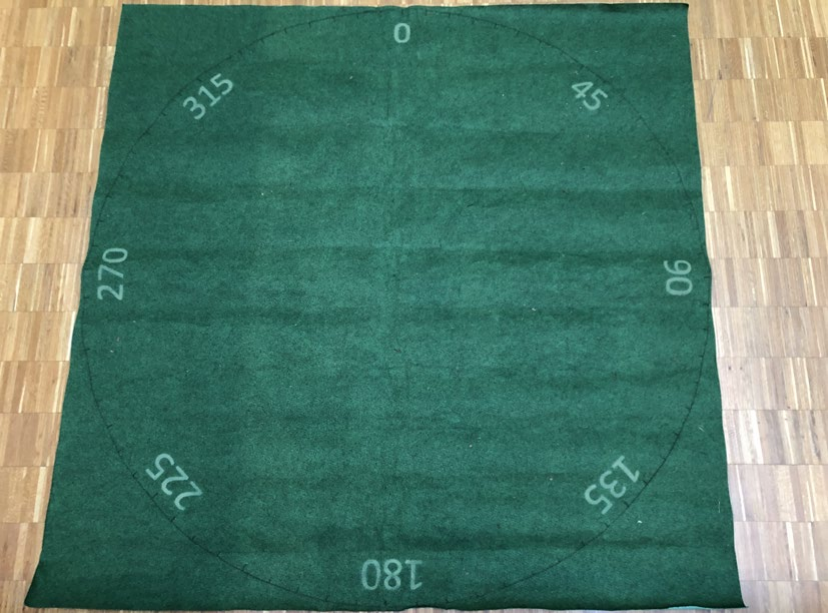
The carpet used for collecting pointing estimates

**Fig. 3:**
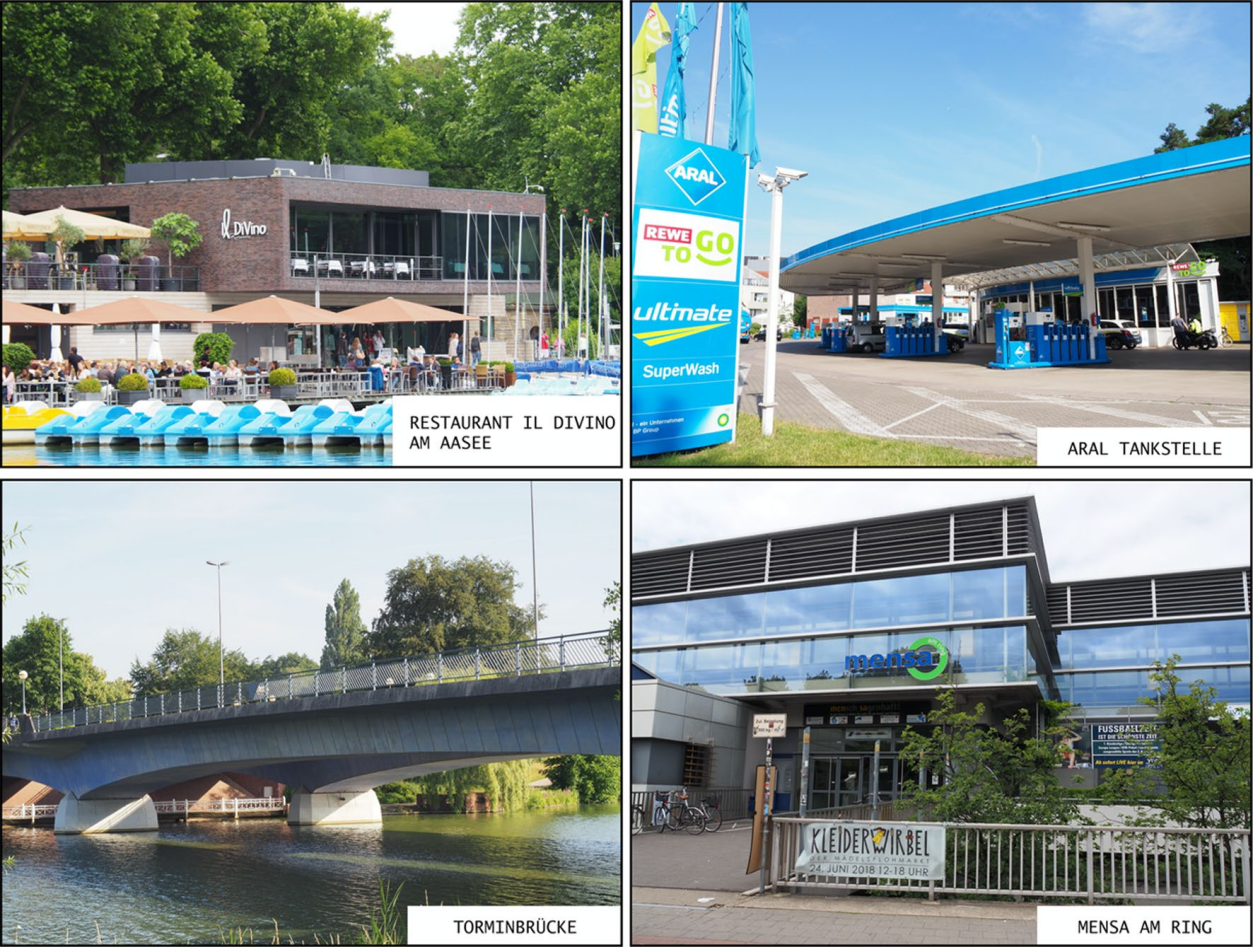
Photographs of known city landmarks that were the targets of estimates in Experiment 1

Each participant had to perform 3 tasks:

(1) *Pointing estimates* In the *real-world* condition, participants were asked to stand in the centre of a green carpet with a white chalk circle (Fig. 2), lying on the sidewalk. The circle contained ticks with numeric indicators ranging from 0$^\circ$ to 315$^\circ$, at 45$^\circ$ intervals. The number 0 on the carpet was oriented towards North, but participants were not explicitly informed about this. The reason for this choice was to provide them with a point of reference (number 0) that is consistent across the tasks. We did not inform about the North direction not to promote the use of allocentric strategies among participants who would not think about or with them spontaneously. Participants had to provide pointing estimates to four distant targets (at a quasi-randomised order). Each target was a known landmark in the city, and none of them were visible from the location of the study. Participants were shown A5-sized photographs of each landmark one-by-one (Fig. 3) and asked to provide pointing estimate verbally using interval numbers visible on the carpet as a guide. Participants were allowed to proceed to the next target if they were not familiar with the current one.

**Fig. 4:**
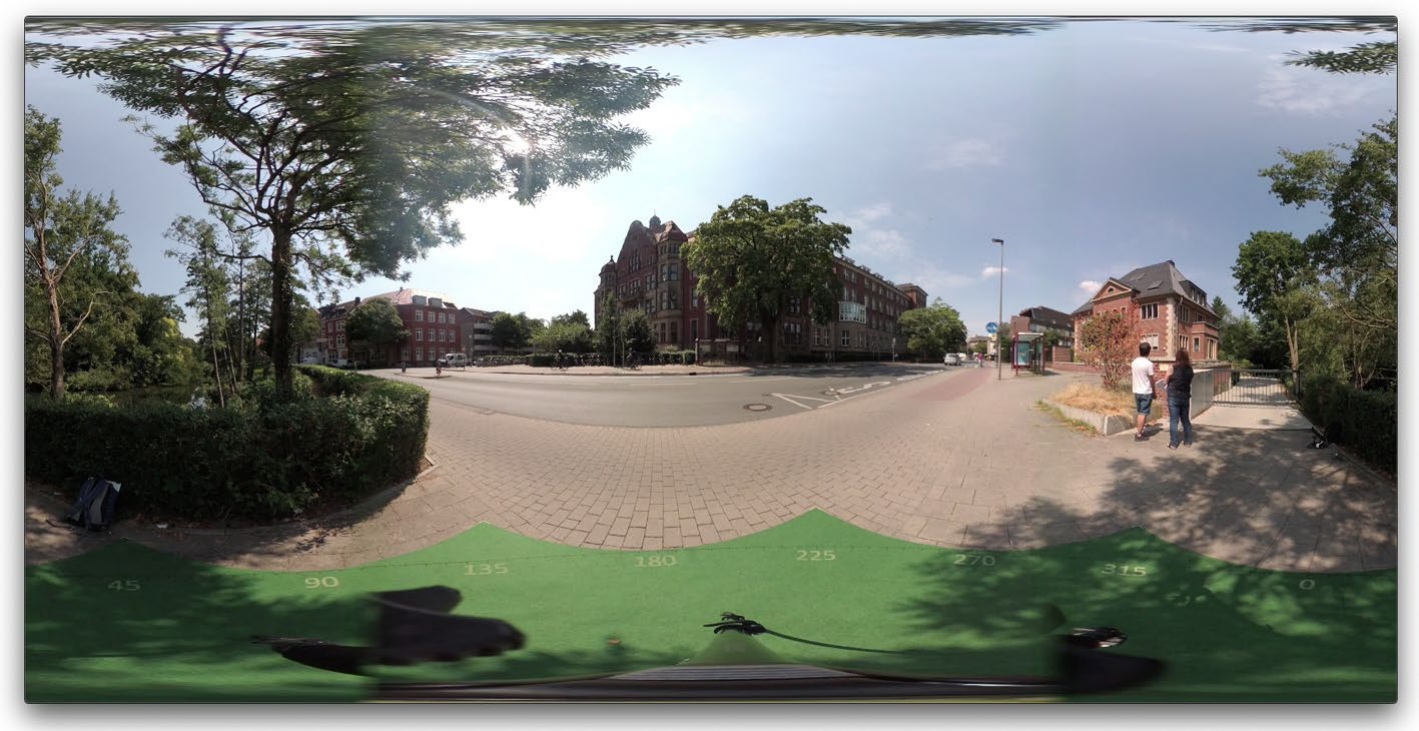
A frame extracted from the 360 video used in Experiment 1. Note that this reproduction distorts the picture that was projected within the head-mounted display. In the experiment, this was experienced as a naturally looking environment surrounding the user who had to rotate their head in order to see the entire frame. The video lasted 5 minutes and played in a loop

In the *VR* condition, a different group of participants was invited to a university laboratory room. They were asked to stand on the carpet (the same as in the *real-world* condition but laying on the laboratory floor) and wear an HTC VIVE virtual reality headset. In the headset, they saw a 360$^{\circ }$ panoramic video recorded at the same location where the *real-world* condition took place (Fig. 4). The camera was mounted on a tripod placed on the carpet, so the carpet was also visible in the panoramic video when the participant looked down. Participants were asked to remove the headset in order to look at the A5 photograph of the target (same photographs as in the *real-world* condition), put the headset back on, and verbally indicate their pointing direction using the carpet visible in the panoramic video for guidance. The set of targets was identical to that in the *real-world* condition and the order was also quasi-randomised.

(2) *Route choice* Directly after making each pointing estimate, each participant was verbally asked which route they would choose if they had to go to that target destination on foot. The experimenter noted down the chosen route. There were 3 routes visible from this location (in Fig. 4: the main street to the left, the main street to the right, and a small street forwards between the buildings visible in the centre-left). This task was identical in both conditions.

(3) *Sketchmapping estimates* Directly after making four pointing estimates and route choices, participants were presented with a single sketchmapping task. The key challenge in analysing sketchmap data is the fact that errors contained in them stem from multiple heterogeneous reasons and intentional schematisations (Montello 2016; Tversky 1992). Thus, we designed a restricted sketchmapping task in which participants’ goal was not to sketch the entire area, but rather to mark presumed positions of targets on a sheet of paper that already contained a pre-drawn sketchmap-like depiction of objects visible from this location. This modification reduces the introduction of errors irrelevant to the goal of our study (i.e., errors other than those coming from the estimation of targets’ locations).

**Fig. 5:**
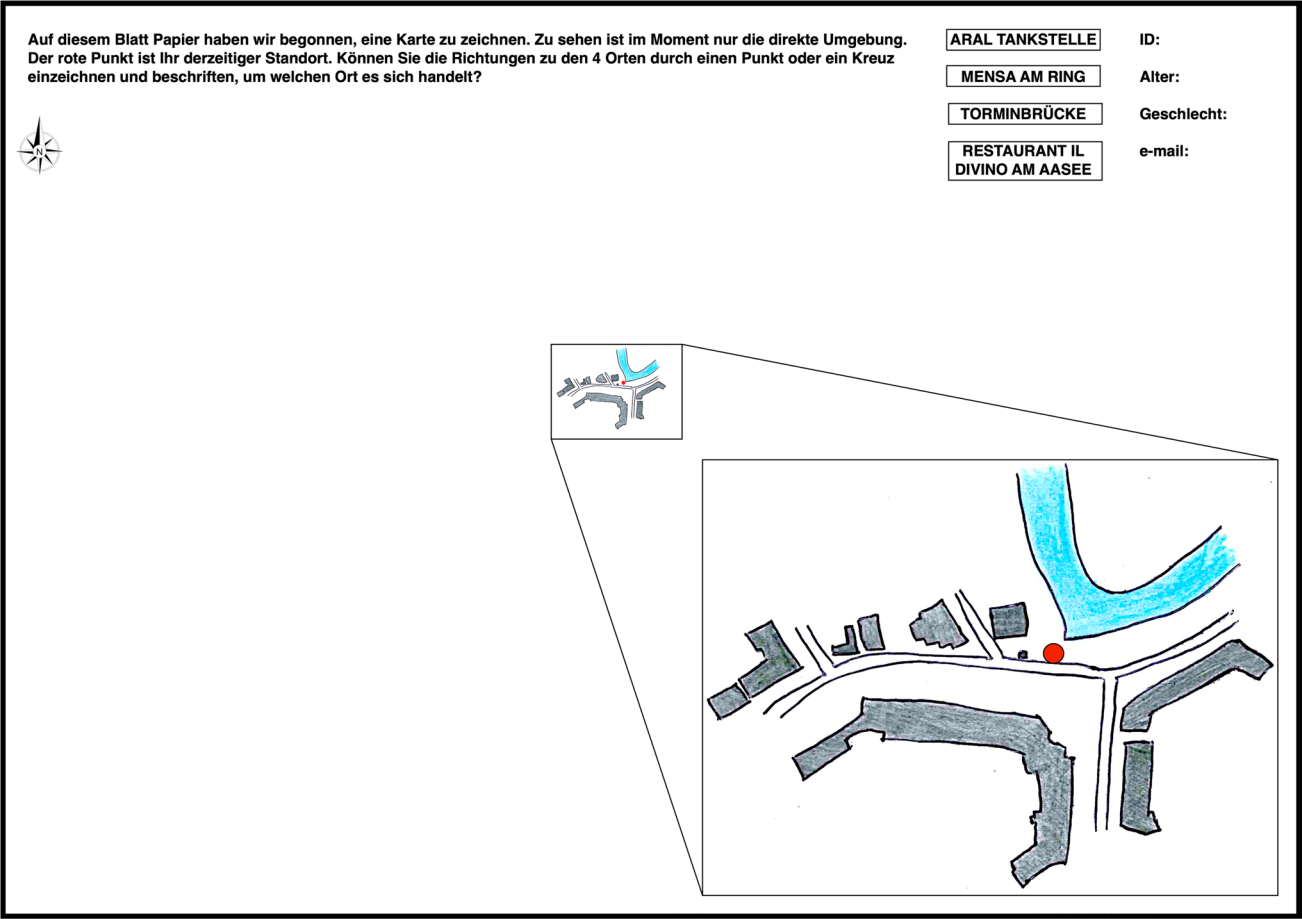
Sketchmap representation of the surrounding (visible) environmental features on which participants performed the sketchmapping task. The red dot indicates participants’ location. Only building and road fragments that were visible from the location were pre-sketched

In our sketchmapping task, participants were shown an A3 sheet of paper with a sketch-like map representation of the surrounding environmental features that were visible from their location (Fig. 5). The drawing was located in the centre of a landscape-oriented A3 paper sheet (the centre is enlarged in the manuscript figure but was not enlarged in the original experimental material). The map was oriented North-up in order to correspond with common views of city maps likely to have been seen by participants in the past (drawing a rotated sketch map could introduce the confounds related to mental rotation abilities). The instruction read (in German): “On this paper sheet we have started to draw a map. Currently, only the direct surrounding can be seen. The red dot indicates your current location. Can you please indicate directions to 4 places by marking a point or a cross and writing down to which place it relates?”. The paper sheet additionally contained an upwards-facing arrow indicating North and fields to write down participant’s ID, age, gender and an optional email address. The page also contained a list of four targets, listed in the top-right corner in the same order as they were queried in the pointing task of the given participant. Participants were asked to mark the estimated position of four targets on the sketchmap. They were allowed to omit targets they were not familiar with (however we did not measure familiarity with each individual landmark beyond unstructured verbal comments). Each target had to be marked with a clear point and a label. The task was identical in the *real-world* and in the *VR* conditions, and in both cases it was performed on a paper sheet.

#### Procedure

Each participant was asked to: (1) provide a pointing estimate to a target and (2) provide a route choice for that target. These steps were repeated until all four targets were queried. (3) Afterwards, participants were asked to provide sketchmapping estimates to all four targets. Participants were free to draw them on a sketchmap at any order of their choosing. After that, the experimenter debriefed and thanked the participant.

### Results

**Table 1:** Summary statistics of Experiment 1

Estimate	Target	Absolute error: M (SD)		Circular error: M (SD)	
		Real world	VR	Real world	VR
Pointing	Aral	25.4 (30.4)	21.5 (26.3)	− 7.4 (39.1)	− 14.5 (30.9)
	ilDivino	29.8 (32.1)	32.5 (26.4)	14.3 (41.6)	21.1 (36.5)
	Mensa	22 (20.1)	20.2 (20.7)	2.4 (29.9)	− 13.9 (25.6)
	Tormin	35.5 (17.7)	30.7 (19.9)	1.1 (40.1)	− 1.2 (37.2)
Sketchmap	Aral	26.9 (21.6)	30.3 (39.4)	− 11.4 (32.9)	5.5 (49.8)
	ilDivino	41.2 (31.7)	45.8 (45.6)	22.8 (47.1)	12.3 (64.1)
	Mensa	19.7 (22.6)	31.8 (47.8)	− 3.7 (30)	− 8.9 (57.1)
	Tormin	44 (24.2)	34.9 (31.2)	16.2 (48.1)	11.9 (45.8)

**Fig. 6:**
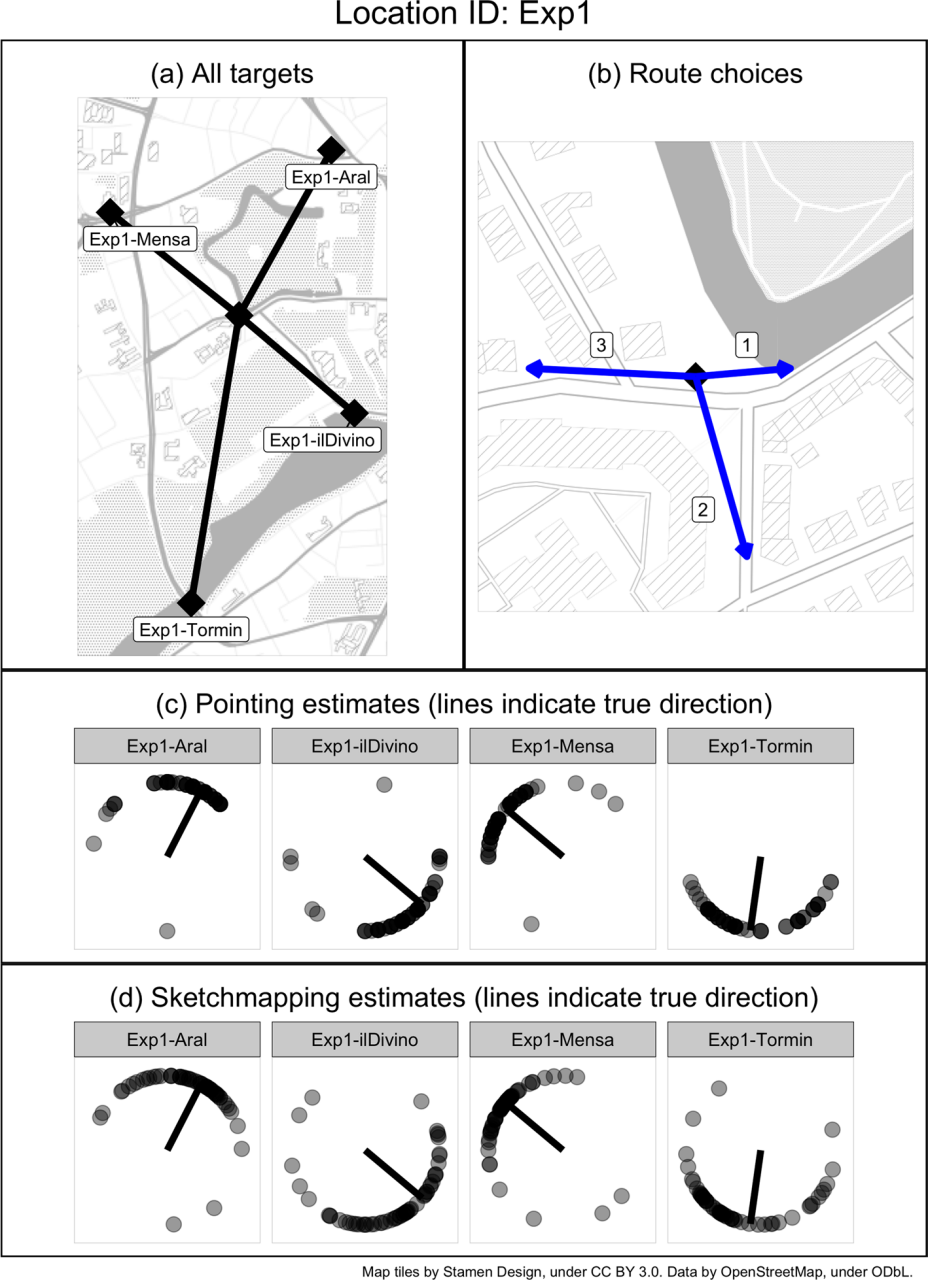
**a** Experimental location in the centre and the location of four targets on the city map. **b** Route choices available from the location. **c** Pointing and **d** sketchmapping estimates made by participants

**Fig. 7:**
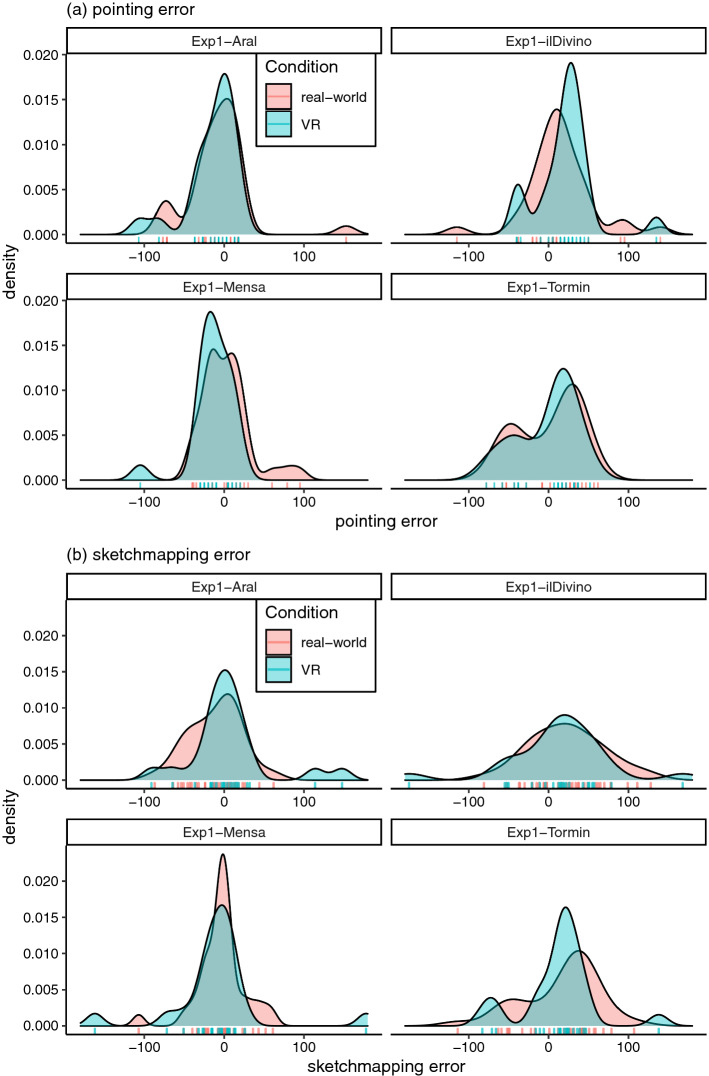
The distributions of **a** pointing and **b** sketchmapping errors collected in the real-world (red) and VR (green) conditions

Table 1 presents summary statistics of the pointing and sketchmapping errors, across the *real-world* and *VR* conditions. Note the difference between the circular and the absolute error: the circular error is centred around 0 (no error), with negative numbers denoting an error to the left, and positive numbers an error to the right. The absolute error ignores the left/right distinction and therefore results in larger mean values. For example, a circular mean value of ($-10$, 10) is equal to 0, while an absolute mean is equal to 10. Statistical analyses that follow use the circular error. The mean of the circular error is the measure of the systematic error, while its standard deviation is the measure of the unsystematic error (Hölscher et al. 2006).

Figure 6 displays the experimental site on a map, together with raw pointing and sketchmapping data. Figure 7 visualises the distributions of pointing and sketchmapping errors across the real-world and VR conditions. We used circular statistics for the validation of the hypothesis, using the entire data shown in Figs. 6 and 7 (and not the summary data reported in Table 1).

As can be seen in Fig. 7, the distributions of data collected in two different conditions are very similar. In order to verify this statistically, we tested a null hypothesis that the *condition* variable (real-world vs VR) does not have an effect on the difference between pointing and sketchmapping estimates. We implemented the Bayesian method of null-hypothesis testing presented by Kruschke (2011) using the brms R package (Bürkner 2017) which is based on Stan (Carpenter et al. 2017), within the framework of Bayesian mixed-effect models (McElreath 2016). We implemented a mixed-effect von Mises distribution model (suitable for circular data) for the effect of *condition* on individual circular pointing errors, with random intercepts across targets and across participants. We implemented a separate, identical model for the effect of *condition* on individual circular sketchmapping errors. We implemented two corresponding null models (with the prior for the effect of the *condition* variable fixed at 0) and derived Bayes factors for the comparison of null model with the unrestricted model. There was strong evidence (BF = 18.30) in favour of the null hypothesis for the pointing errors and strong evidence (BF = 12.12) in favour of the null hypothesis for the sketchmapping data.

Based on the visual investigation (Fig. 7) and on this statistical result, we concluded that VR is a valid substitute for collecting data in the real world, in this experimental procedure. This result confirms Hypothesis 1.

In order to investigate whether gender unbalance between the two groups in our sample confounded the above result we implemented additional models using declared *gender* as the independent variable instead of *condition*. The null hypothesis was that there was no influence of gender on the results. There was strong evidence (BF = 11.63) in favour of the null hypothesis for the pointing errors, and moderate evidence (BF = 5.34) in favour of the null hypothesis for the sketchmapping data. This indicates that there was no effect of gender on the pointing and sketchmapping estimates in our sample.

## Experiment 2

We hypothesised that pointing to/sketchmapping of distant targets will be biased in the direction of the chosen route. In order to test this hypothesis, we designed a study in which a new group of participants was asked to point to, choose a route to, and sketch the location of four different targets from each of six locations in the city of Muenster, Germany. We designed location-target pairs so that between 1-3 targets from each location were located across a barrier—making it impossible to choose a route that would lead directly towards the target.

### Methods

Following the open science principles, we declare this manuscript as *exploratory research* (within the framework of Wagenmakers et al. (2012)) and as a *postdiction analysis* (within the framework of Nosek et al. 2018). Our research questions and hypotheses were formulated before collecting the data, but the analysis code was written afterwards.

#### Participants

We recruited 29 participants (11 males and 18 females; mean age = 25.50, SD = 3.80; mean years lived in the city = 6.80, SD = 6.60) using university mailing lists and participant recruitment systems at the University of Muenster, Germany. Participants had to sign informed consent forms following the study’s ethics clearance from the institute’s ethics committee and they were paid 10 euro per person. The experiment lasted about an hour. Sample size determination was opportunistic, restricted by the available funds. We account for the limited sample size in the statistical analysis by employing the Bayesian approach and multi-level statistical modelling that has higher statistical power due to utilising information from all multiple observations collected per participant.

#### Material

**Fig. 8:**
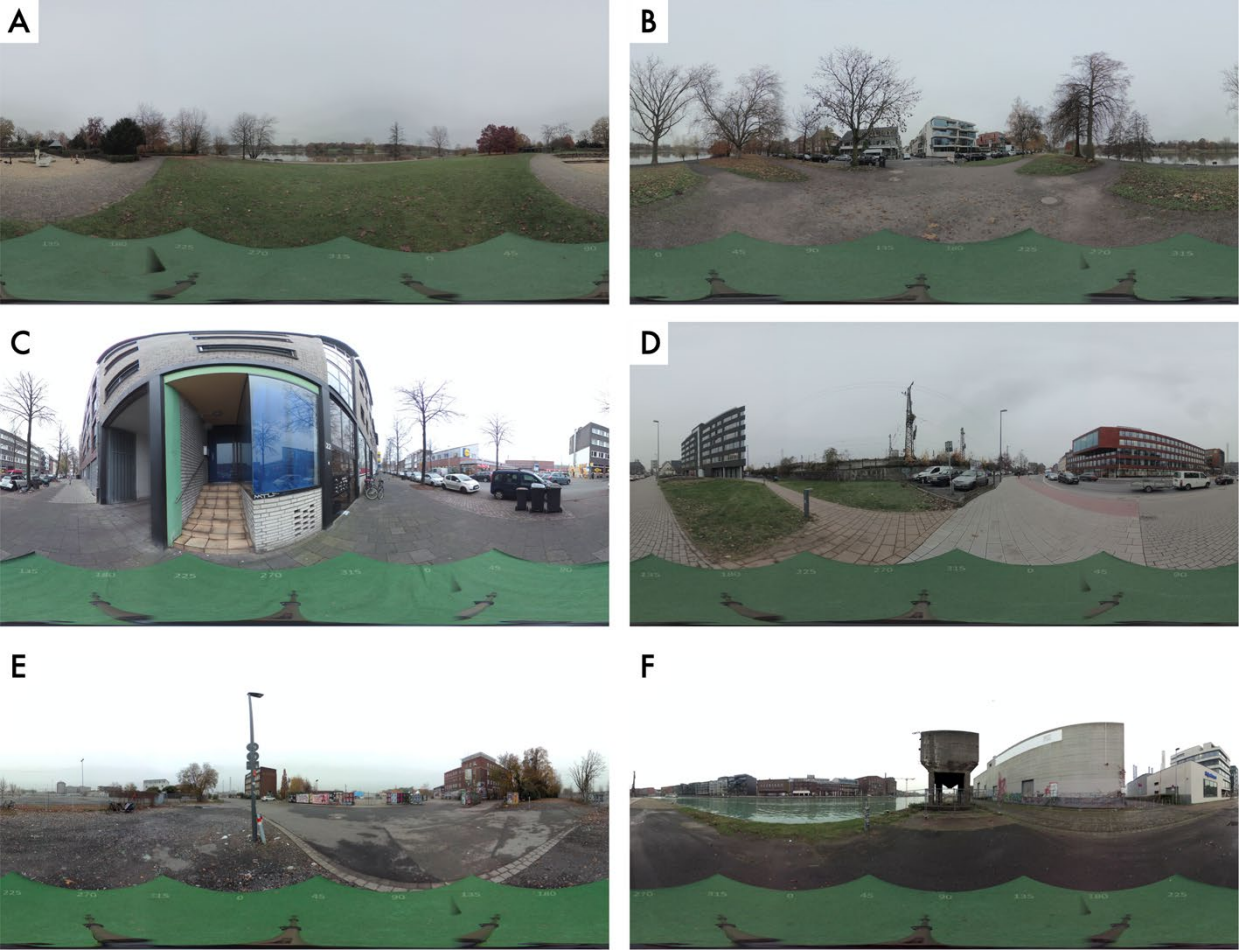
Panoramic photographs of the locations used in Experiment 2. Pictures of locations E and F have been brightened for the presentation in the manuscript but their visibility was flawless in the HTC VIVE headset

**Fig. 9:**
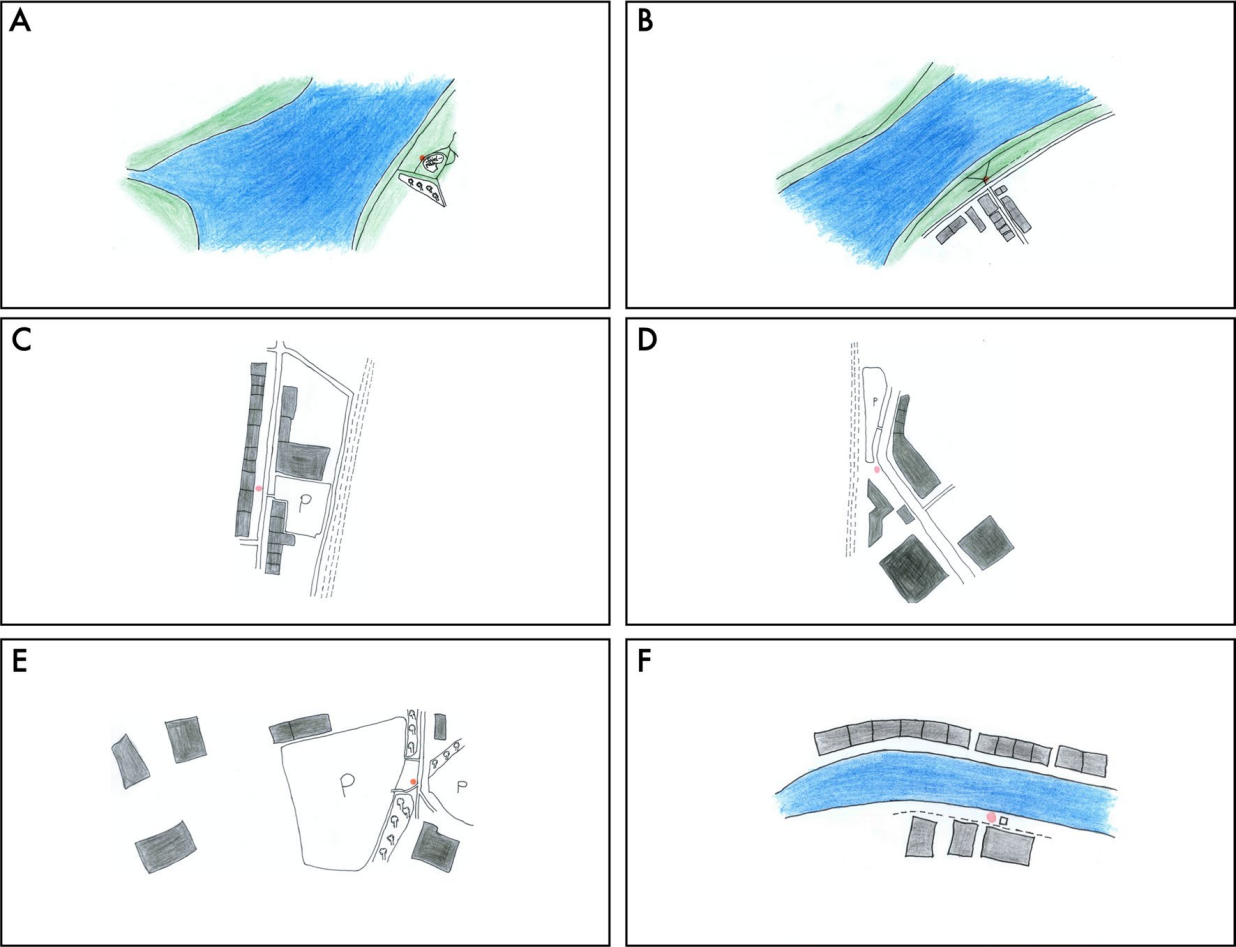
Sketchmap depictions presented in the centre of A3 paper sheets used in the sketchmapping task of Experiment 2

The experiment was conducted exclusively in the Virtual Reality setting. Materials were prepared according to the same template used in the VR condition of Experiment 1. There were six locations and four targets from each location. In further descriptions we denote location IDs from Experiment 2 with upper-case letters “A-F”, and each target with its popular name. The letter ID proceeding each target’s name (e.g. “A-”) is the identifier of the location from which this target was queried (targets from Experiment 1 are identified in the dataset files with the prefix “Exp1-”). All locations were presented in the HTC VIVE headset. Instead of the looped video as in Experiment 1, participants saw high-quality panoramic photographs (Fig. 8). Photographs of targets were triggered by the experimenter to appear within the virtual reality environment (and not shown on print-outs, as in Experiment 1). Thanks to this modification of the experimental procedure, participants only had to remove their headset once per location—in order to provide the sketchmapping estimates on an A3 paper sheet (Fig. 9).

#### Procedure

The procedure was identical to the one employed in the *VR* condition of Experiment 1. Locations were presented to participants in a randomised order. The order of targets within each location was quasi-randomised. Participants performed all tasks (i.e., the pointing estimate, route choice, and sketchmapping estimate) from a single location before being presented with the next location. In total, each participant could provide pointing estimates, route choice, and sketchmapping estimates to 6 x 4 = 24 targets. Participants could skip to the next target (or entire location) if they verbally indicated not being familiar with the current target (or location).

#### Data analysis

Missing data

With this number of participants and tasks, the dataset contained 696 potential pointing estimates, 696 route choices, and 696 sketchmapping estimates. However, there were 109 pointing estimates missing, 109 route choices missing, and 112 sketchmapping estimates missing. Missing data occurred when participants were not familiar either with the location or with the target, or when they made sketching mistakes (e.g. marking the same target on the sketchmap twice). Our method did not differentiate between nor recorded the reasons for which one would skip a trial. The reported analyses include 9 estimates during which participants verbally indicated that they are not sure where a landmark is but tried to provide an estimate nevertheless. In total, we obtained 587 pointing estimates, 587 route choices, and 584 sketchmapping estimates.

##### Calculating angular deviation of routes

In order to link the property of the route leading towards a target with the corresponding pointing and sketchmapping errors, we calculated the angular deviation between the initial segment of the chosen route and the true direction to each target. This means that each route segment that was available to the participant as a route choice option (e.g. there are three such segments at the location presented in Fig. 6b) would receive four “angular deviation” values, describing its angular deviation to each of four targets. For instance, in Fig. 6, the angular deviation of segment “1” to the target “Aral” is 66$^{\circ }$, to “ilDivino” is − 38$^{\circ }$, to “Mensa” is 144$^{\circ }$, and to “Tormin” is − 95$^{\circ }$. Appendix A contains a table listing all combinations.

The value that was ultimately correlated in our statistical models with the pointing/sketchmapping estimate of the given target depended on the route option chosen by each individual participant. For instance, using the illustration from Fig. 6, if a participant chose segment “3” as the preferred route to “Aral”, the angular deviation value used in the analysis would be -116$^{\circ }$, which is the angular deviation between segment “3” and “Aral” (as noted in Table 2 of Appendix A). Note that this method classifies routes based on their initial segment only. The choice of this approach is based on the known evidence for the importance of initial route segments in navigational decision making (Bailenson et al. 2000; Dalton 2003; Wiener and Meilinger 2018). It is likely, however, that this variable will not always be representative of how large a detour the entire route takes. It is possible to extract different properties of routes in order to distinguish those that lead around a barrier from those that lead relatively directly to the target. We have conducted an additional analysis of two such alternative measures and report them in Appendix B.

##### Distinguishing uniform and varied path choice cases

Exploratory data analysis revealed that replicating the pattern observed accidentally by Schwering et al. (2017) in alternative locations is difficult: For many cases, despite the existence of a barrier that could be bypassed from two alternative directions, almost all participants consequently selected a single initial route segment (we observed that 11 out of 24 location-target pairs were dominated by a single choice with either none or only one participant making a different decision). This called for a different statistical approach than initially envisioned. Thus, we divided the dataset into two parts and applied two separate statistical approaches to each of them:

- Part 1 consists of those location-target pairs where at least two initial route segments were chosen by at least 5 participants and is further referred to as *varied path choice* location-target pairs. Such cases are conceptually closer to the prototypical case observed by Schwering et al. (2017). In our analysis, there were 4 location-target pairs that had a *varied path choice*. Although they are rare in the dataset, *varied path choice* allows us to make comparisons within single locations, based on two path choices that were similarly optimal.
- Part 2 consists of those location-target pairs where all or almost all participants selected a single, same route segment. We refer to this part as *uniform path choice* location-target pairs and define them as situations where only one initial route segment was selected by more than 5 participants. In our analysis, there were 20 location-target pairs that had a *uniform path choice*.

##### Data exclusions

The analysis ignored cases where participant chose one of the routes selected by less than 5 other people. This step ensures that our investigation of the systematic bias in pointing and sketchmapping estimates focuses on *already well-performing participants*. There are many potential (non-systematic) biases that can be involved in estimates made by participants who were wrong about the path choice (for instance, they could have had a consistently rotated mental representation of the entire environment (Meilinger et al. 2016)). After removing these cases, the data on which the statistical models for the *varied path choice* cases were fit consisted of 94 pointing estimates, and of 94 sketchmap estimates. The data on which the statistical models for the *uniform path choice* cases were fit consisted of 450 pointing estimates, and of 446 sketchmapping estimates. In sum, our analyses exclude 43 out of 587 pointing estimates and 42 out of 584 sketchmapping estimates that presumably come from the most disoriented participants. Our analyses can be repeated after altering these exclusion criteria using the instructions in the provided data and code repository.

### Results

#### Varied path choice cases

In order to examine Hypothesis 2 within the *varied path choice* location-target pairs, we divided responses into those where a participant chose an initial route segment deviating to the left (w.r.t. the true direction to the target) and those where a participant chose the initial route segment deviating to the right. We tested whether participants who chose left-deviating routes had more left-deviating pointing/sketchmapping errors, compared to participants who chose right-deviating initial route segments. Note that within the *varied path choice* location-target pairs, both left- and right-deviating route segments were plausible alternatives, thus they are unlikely to systematically differentiate participants by their knowledge of the city.

**Fig. 10:**
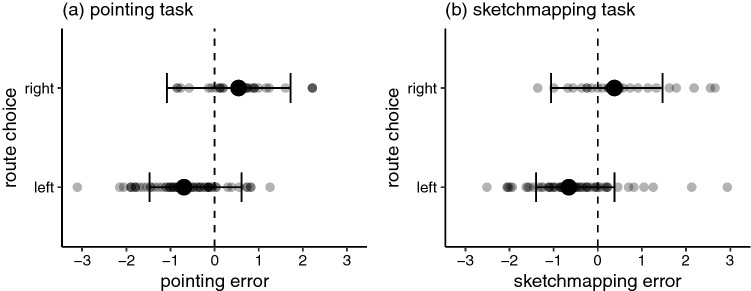
Visualisation of the model fitted to the relation of the route choice and the **a** pointing and **b** sketchmapping task results. Points indicate raw data. Larger dots indicate the mean. Models are fitted on a (-pi, pi) scale

In order to test Hypothesis 2 statistically, we evaluated whether pointing to/sketchmapping of distant targets was be biased in the direction of the chosen route. We implemented the Bayesian method of null-hypothesis testing presented by Kruschke (2011). We implemented two mixed-effect von Mises distribution models for the effect of a categorical variable *route choice* on individual circular errors (one model for pointing errors and another model for sketchmapping errors). Random effects included random intercepts across targets and across participants as well as random slopes for the effect of *route choice* within targets. We implemented two corresponding null models (with the prior for the effects of *route choice* fixed at 0) and derived Bayes factors for the comparison of null model with the unrestricted model. There was moderate evidence (BF = 5.07) in favour of Hypothesis 2 for the pointing errors, and moderate evidence (BF = 3.20) in favour of Hypothesis 2 for the sketchmapping data. Tables 2 and 3 present the estimates of the models. A positive estimate of the *route choice (right-vs-left)* parameter indicates that choosing the route deviating to the right was associated with pointing/sketchmapping errors more to the right (i.e., positive errors), compared to choosing the route deviating to the left. Figure 10 displays these results visually. This figure also shows that the distribution of pointing and sketchmapping errors is bimodal (i.e., clustered in two groups, depending on the route choice). This situation is different from a potential alternative in which the errors made would be normally distributed around the true direction to the target.

In order to test whether these results were affected by participants’ overall familiarity with the city, we implemented models corresponding to the ones above, but containing an additional variable of years spent in the city. Its values ranged from 2 to 30 years, with *M* = 6.83 and SD = 6.61. There was very strong evidence (BF = 1/61.85) against the influence of years spent in the city on the pointing errors, and extreme evidence (BF = 1/128.04) against the influence of years spent in the city on the sketchmapping errors.

Our results confirm Hypothesis 2 for the *varied path choice* cases: pointing and sketchmapping errors were more likely to the left, if participant chose the left-deviating route from the available plausible alternatives and more likely to the right, if participant chose the right-deviating route. The estimated effect of that difference corresponds to 56 degrees of difference between left and right group for the pointing data and 49 degrees of difference for the sketchmapping data.

**Table 2:** Posterior mean, standard error, and 95% credible interval for route choice parameter of the pointing error model for the varied path choice cases

Parameter	Mean	SE	Lower bound	Upper bound
Intercept	− 0.35	0.29	− 0.91	0.32
Route choice (right vs left)	0.63	0.26	− 0.02	1.11
Kappa	3.01	0.46	2.20	4.01

**Table 3:** Posterior mean, standard error, and 95% credible interval for route choice parameter of the sketchmapping error model for the varied path choice cases

Parameter	Mean	SE	Lower bound	Upper bound
Intercept	− 0.34	0.24	− 0.84	0.19
Route choice (right vs left)	0.52	0.27	− 0.08	1.03
Kappa	2.22	0.31	1.66	2.87

#### Uniform path choice cases

**Fig. 11:**
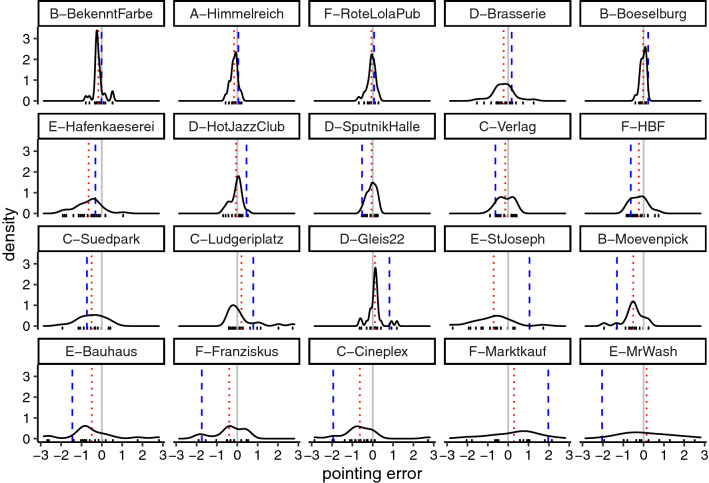
Raw data from the pointing task. Only uniform path choice cases for routes that gathered more than 5 choices are plotted. The grey solid line indicates 0 (no pointing error), the red dotted line indicates circular mean, and the blue dashed line indicates the angle of the initial route segment to the given landmark. Note that for cases where the initial route segment was deviating more from the true direction (lower rows of the figure—blue dashed line further away from grey solid line), participants’ pointing tended to be biased in the same direction (red dotted line was on the same side of the grey solid line as the blue dashed line)

In order to test Hypothesis 2 statistically, we evaluated whether pointing to/sketchmapping of distant targets was biased in the direction of the chosen route. Within the *uniform path choice* location-target pairs, we tested whether a larger angular deviation of the initial route segment chosen by a given participant was associated with a larger circular error *in the same direction* (left or right) in pointing to/sketching the given target.In other words, the question being answered was: are those location-target pairs that have less direct routes towards the target associated with poorer pointing/sketching and is the direction of the errors consistent with the direction of the initial route segment?

Figure 11 visualises the raw pointing data grouped by separate targets. The figure is ordered by the absolute angular deviation of the initial route segment so that the lower two rows of the figure show cases where participants pointed to targets, towards which only indirect routes were available. It can be seen that as the deviation of the initial route segment grew, participants’ mean pointing tended to be biased in the same direction—the blue dashed line and the red dotted line tended to stay on the same side of the grey solid line.

In order to statistically test whether this patter generalises across the dataset, we examined a hypothesis that there is a circular–circular correlation between the angle of the path chosen by the participant and the pointing/sketchmapping circular errors. We implemented the Bayesian method of null-hypothesis testing presented by Kruschke (2011). We implemented two mixed-effect von Mises distribution models for the effect of sine- and cosine-transformed *angular deviation of the chosen route* on individual circular errors (one model for pointing errors and another model for sketchmapping errors). The sine- and cosine-transformations ensure that the distribution of the predictor variable is wrapped, respecting the fact that $-179^{\circ }$ is only 2$^{\circ }$ apart from +179$^{\circ }$ (Sarma and Jammalamadaka 1993). Random effects included random intercepts across targets and across participants as well as random slopes for the effect of sine- and cosine-transformed *angular deviation of the chosen route* within participants. We implemented two corresponding null models (with the prior for the effects of sine- and cosine-transformed *angular deviation of the chosen route* fixed at 0) and derived Bayes factors for the comparison of the null model with the unrestricted model. There was strong evidence (BF = 1/10.49) against Hypothesis 2 for the pointing errors and very strong evidence (BF = 1/85.51) against Hypothesis 2 for the sketchmapping data. Table 4 presents the estimates of the pointing error model and Table 5 the estimates of the sketchmap error model (note that the sine- and cosine-transformed estimates are not intuitively interpretable).

**Fig. 12:**
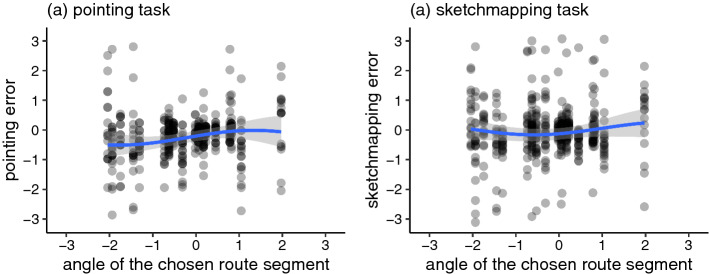
Visualisation of the model fitted to the relation of the angle of the initial route segment and the **a** pointing and **b** sketchmapping task results. Points indicate raw data. Models are fitted on a (-pi, pi) scale

Figure 12 shows the raw data (grey points) and the fitted models (blue lines with grey 95% credible interval). As visible, the raw results of the pointing task (grey points) cluster around a diagonal line, but the slope of this line is not significantly different from a null effect. These results reject Hypothesis 2 for the uniform path choice cases: pointing and sketchmapping errors were not more likely to the left, if the route chosen towards the target deviated to the left and were not more likely to the right, if the route chosen towards the target deviated to the right.

**Table 4:** Posterior mean, standard error, and 95% credible interval for each parameter of the pointing error model for the uniform path choice cases

Parameter	Mean	SE	Lower bound	Upper bound
Intercept	− 0.13	0.08	− 0.29	0.03
Sin(route angle)	0.12	0.06	0.01	0.23
Cos(route angle)	0.03	0.10	− 0.16	0.21
Kappa	3.85	0.26	3.37	4.37

**Table 5:** Posterior mean, standard error, and 95% credible interval for each parameter of the sketchmap error model for the uniform path choice cases

Parameter	Mean	SE	Lower bound	Upper bound
Intercept	0.03	0.06	− 0.10	0.16
Sin(route angle)	0.06	0.06	− 0.06	0.19
Cos(route angle)	− 0.09	0.08	− 0.26	0.07
Kappa	2.51	0.15	2.22	2.81

## Discussion

**Fig. 13:**
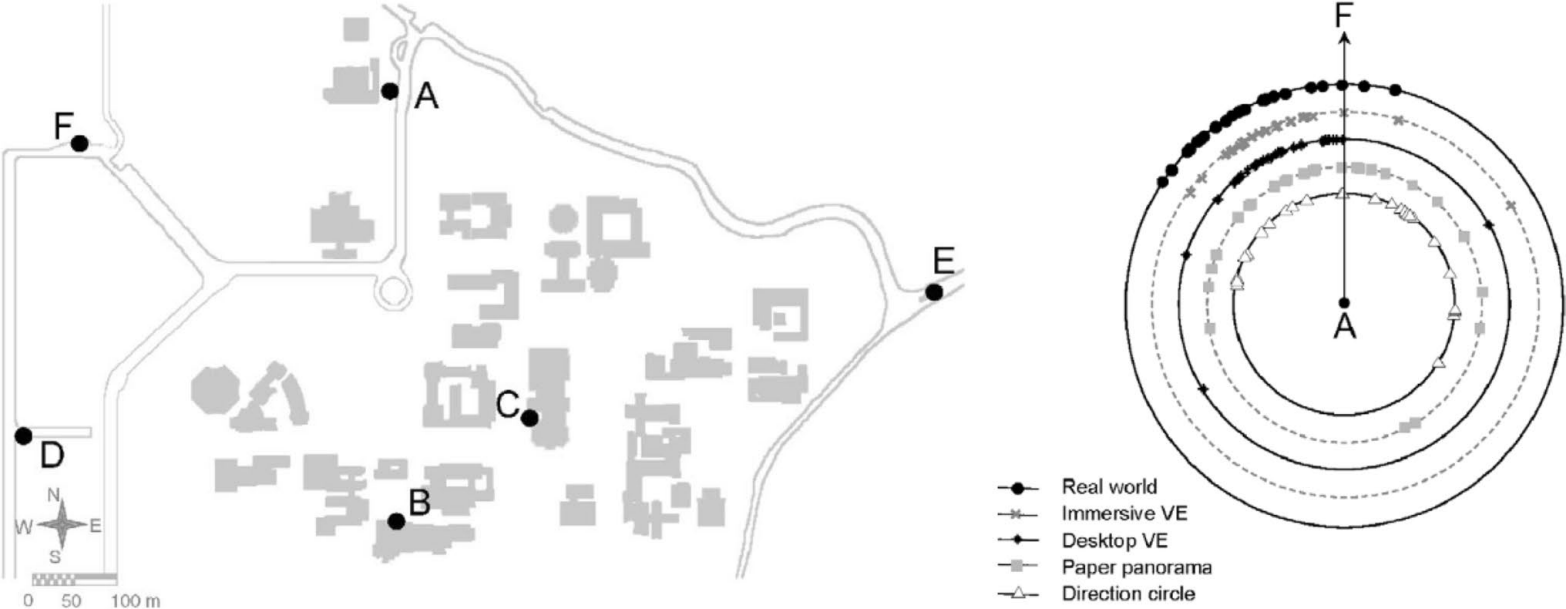
The environment (left) and the pointing estimates (right) from location A to F that would require a leftward de-tour, obtained by Waller et al. (2004). Reproduced with permission

This study provided preliminary evidence that in some urban situations (namely: when two plausible but indirect route alternatives are available around a large barrier) there is a systematic bias in pointing/sketchmapping performance associated with the preferred route choice. The angle of the initial route segment chosen by the participant was correlated with the pointing errors they make. The evidence for this bias was stronger for pointing, than for sketchmapping data. The experimental procedure relied on replicating earlier accidental finding by Schwering et al. (2017), in new urban locations. Investigating a corresponding effect in urban locations with only one plausible navigational choice showed no systematic bias in pointing and sketchmapping errors. It therefore seems that a systematic bias in pointing and sketchmapping to distant targets is moderated by the presence of multiple competing route alternatives, but the question why this happens remains open to future investigation. One tentative explanation would be the fact that in the presence of multiple plausible alternatives each participant takes into account information about the direction of both paths, even if they have a strong preference for one of them. A situation in which only one plausible path is available could be evaluated with a different cognitive strategy that is not susceptible to the bias. For example, encountering a situation with two plausible paths could promote thinking about these two options as categories (left vs right) and cause the pointing estimate to bias towards the category’s prototype (Huttenlocher et al. 1991; Waller et al. 2004).

These findings make it possible to identify some situations in experimental design of in situ spatial cognition experiments that might cause systematic biases in survey knowledge estimates. When direct movement towards the queried target is not possible from the current standpoint, and two equally plausible routes are possible, there is a risk of systematic bias in pointing and sketchmapping errors, consistent with the direction of the preferred route choice. Our study highlights the pressing importance of considering such situations in the experimental design of urban-based spatial cognition studies. Specifically, researcher choosing the location from which survey knowledge is queried should consider the visibility of routes to the target of pointing and sketchmapping tasks. Otherwise, results of survey knowledge estimates obtained from different locations in the city might be biased to different degrees. One possible example of such a situation may be the data presented by Waller et al. (2004). As reproduced here in Fig. 13, data from their paper show a leftwards bias in pointing estimates from location A to landmark F. Comparing this with the map of the environment suggests that this bias (unexplained by the authors) would fit the suggested tentative explanation: there are two plausible routes available from A to F (although the one heading North is cropped in the original figure). The authors provide a discussion as to why the bias is not observed in the *panorama* and *direction circle* conditions suggesting that the property of the stimulus might provide or highlight axes (categories) that bias the pointing estimate. They suggest that the axes provided by the *direction circle* method are different compared to the first-person view of the environment. According to the authors, despite providing such a perspective, the paper *panorama* method does not facilitate the presentation of ground-level information in a way comparable to more realistic computer simulations or real-world experience (Waller et al. (2004)). A possibility not discussed by the authors but relevant in the context of our data is that immersive first-person view methods may be more directly accessing the perceptuo-motor information about actions and experiences in the environment (Brunyè et al. 2012; Sadalla and Montello 1989; Wang et al. 2012, 2020) such as the possible behaviour of walking to the left or to the right.

To our knowledge, this study is the first to show a systematic route effect in pointing and sketchmapping estimates. This is in line with previous literature that showed that route effects exist in distance estimates (Klippel et al. 2005; Lederman et al. 1987; McNamara et al. 1984). Both distance and direction estimates are manifestations of survey knowledge, so the joint evidence converges on the assumption that available routes distort survey knowledge. The novel evidence from our study is that route effects are different in different urban configurations and might be stronger when multiple plausible routes to the target are available.

In the presented experiment, sketchmapping bias was smaller compared to the pointing bias. This might be due to the specific implementation of the sketchmap task in our study. First, our material included some pre-drawn elements that provided spatial context around the location. We included only those elements that were actually visible from the location, but seeing them in the allocentric view might make it easier to infer other spatial relations. Second, the sketchmapping task was always performed after the pointing, which might make it easier to refine estimates the participant has already made in the previous task (although no feedback was given between the tasks). Moreover, it may be easier to make survey knowledge estimates in a sketchmapping task due to the properties of this task itself. While sketchmapping, participants can continuously cross-check their individual estimates against other estimates provided in the sketch. This allows them to seek for the most subjectively satisfying solution with respect to all targets. It also promotes the use of additional information about spatial relations between targets (Montello 1991). As such, sketchmapping might not reveal systematic distortions that are present for some—but not all—landmarks in the sketch. Some characteristics of the pointing task make it more prone to biases investigated in this study. First, it is performed one-by-one, so each pointing can made (and potentially biased) independently from the previous one. This can promote larger biases in an experimental set-up like ours where participants’ knowledge of each landmark’s location is heterogeneous—one pointing might be therefore much more erroneous than the previous one. It is important to note that this is *not* a characteristic of all spatial cognition experiments; in many, participants are asked to point to landmarks that they know similarly well. Future work aimed at explaining the observed difference in biases between pointing and sketchmapping tasks should control multiple factors that differentiate the tasks beyond the ego- and allocentric perspective of the queried representation. These include task order, the sequential vs simultaneous display of landmarks that are queried, making the North orientation explicit or not, and providing visual information on the sketch that is comparable to what is seen from the egocentric perspective during pointing.

It is important to acknowledge that an intrinsic feature of using natural environments for spatial cognition studies is the fact that they combine multiple sources of various bias. It might be impossible to ever isolate all of them. The role of studies such as ours is to raise awareness about types of experimental situations that might likely cause systematic biases but not necessarily to fully eliminate them. Their deeper understanding is a long-term task for the discipline that can be greatly facilitated with the methods of cumulative open science. As researchers generate more data in in situ spatial cognition studies (for various research questions), assumptions about the existence of systematic biases could be formally tested in mega-analyses (Koile and Cristia 2021)—an alternative to meta-analysis that uses unaggregated raw data from past studies. However, as for now too little datasets in the field are open. One solution already available for dealing with the issue of systematic in situ biases is to ensure sufficient diversity of natural environments in a study—for instance, with respect to the amount and direction of plausible route alternatives visible from the location of pointing.

## Limitations

Our experiment did not counter-balance task order. Participants always had to point to four targets (one by one) first and only then to sketch them on a sketchmap. Therefore, pointing to a target always proceeded sketching it. Smaller bias in sketchmapping estimates (compared to pointing estimates) that is visible in our results should not be interpreted as conclusive.

Our method allowed participants to skip a response, but did not record the reasons for doing so, nor did it quantify familiarity with individual landmarks. This approach limits the precision of measurement. For example, participants could differ in their threshold of certainty under which they would decide to skip a trial. A systematic bias also cannot be ruled out, for example if most participants had a relatively low threshold for skipping a trial (i.e., if only slightly uncertain of where a landmark is, they would decline to provide an estimate of its direction). Thus, only responses with high certainty would be included in the dataset. Informal conversations with our participants suggest that this was not the case and that participants tried to make an estimate unless they did not know the target at all or were completely disoriented. Analyses including “years spent in the city” as an indicator of overall familiarity with the environment showed no influence on the statistically significant results, but this variable might be a poor predictor of familiarity with landmarks queried in the experiment. Future studies should quantify familiarity with individual landmarks or use sampling that ensures similar familiarity among participants.

We also emphasise the exploratory character of the study. The distinction of uniform and varied path choice cases was difficult to plan for, because we could not predict the distribution of route choices from each location used in the study. An extension of this work could involve a virtual environment where participants have to learn possible routes to the destination in a controlled manner, before providing their route choice, pointing, and sketchmapping estimates.

## Conclusions

This paper showed that in some urban situations, direction estimates from pointing and sketchmapping tasks in an urban environment are biased in the direction of the route chosen to reach the respective target. Previously published data of Waller et al. (2004) and Schwering et al. (2017) have shown consistent biases in some instances of their pointing task but did not explain them. The current manuscript provides first explicit evidence for the existence of “route effects” in direction estimates that have previously been shown in distance estimations.

This work has theoretical implications for spatial cognition studies by providing another evidence for the cognition–action link in human understanding of spatial environments (Barsalou 2008; Tversky 2009; von Stülpnagel and Steffens 2012). It also has methodological implications supporting concerns of other researchers with regard to the choice of wayfinding routes in urban-based spatial cognition studies (Mazurkiewicz et al. 2020). Our results suggest that route choices might introduce systematic biases, making some findings of such studies non-replicable in new environments.

Lastly, our work has practical implications for cartography and spatial human–computer interaction (Galvão et al. 2021, 2022; Krukar et al. 2020), as it shows how to identify spatial context particularly challenging for human survey knowledge estimation. Wayfinding support applications could consider spatial context in which two equally plausible routes are available to a distant target and provide their user with additional support in these challenging situation.

## Supplementary Information

Below is the link to the electronic supplementary material.

Supplementary file 1 (pdf 1117 KB)

Supplementary file 2 (pdf 707 KB)

## Data Availability

All data and code used to produced this paper can be accessed via the following link: https://osf.io/8mnzv/ The manuscript was generated using the papaja R package (Aust and Barth 2022).
