# Supplementary material for: Route effects in city-based survey knowledge estimates
Source: Cogn Process. 2023 Jan 23;24(2):213–31. doi: 10.1007/s10339-022-01122-0 (PMC10110726; doi:10.1007/s10339-022-01122-0)
Supplement: Supplementary file 1 — (pdf 1117 KB) [file 10339_2022_1122_MOESM1_ESM.pdf]

Appendix A

anonymised

Summary statistics

Table 1: Average pointing and sketchmapping errors to all locations analysed in Experiments 1 and 2.

		Absolute error		Circular error	
location	landmark	pointing: M (SD)	sketchmap: M (SD)	pointing: M (SD)	sketchmap: M (SD)
A	A-Brillux	55.07 (37.77)	63.66 (37.97)	-45.07 (49.63)	-35.31 (65.93)
	A-Himmelreich	23.3 (41.44)	36.91 (45.94)	4.96 (47.54)	-7.73 (58.95)
	A-Sparkasse	55.65 (41.57)	49.96 (44.96)	-20.17 (67.38)	-7.04 (67.63)
	A-Zoo	43.57 (37.28)	40.82 (38.31)	15.57 (55.74)	14.32 (54.62)
B	B-BekenntFarbe	20.17 (28.46)	18.62 (35.33)	-15 (31.59)	-5.17 (39.74)
	B-Boeselburg	27.52 (50.8)	31.52 (47.75)	-9.2 (57.29)	-13.28 (55.96)
	B-Moevenpick	42.58 (37.93)	40.79 (38.95)	-19.33 (54.23)	-18.88 (53.68)
	B-Wasserturm	46.65 (32.41)	44.2 (40.41)	-6.75 (57.39)	-14.3 (58.94)
C	C-Cineplex	47.76 (45.69)	57.12 (51.99)	-32.48 (58.01)	-23.96 (74.22)
	C-Ludgeriplatz	33.92 (40.71)	36.52 (39.84)	3.68 (53.31)	2.12 (54.51)
	C-Suedpark	44.09 (35.41)	43.81 (43.99)	-7 (56.91)	4.29 (62.7)
	C-Verlag	24.89 (24.51)	33.79 (46.37)	-1.53 (35.39)	-6.21 (57.57)
D	D-Brasserie	24.68 (22.6)	24.37 (27.74)	-12.89 (31.14)	0.89 (37.22)
	D-Gleis22	19.25 (30.48)	25.33 (34.92)	11.46 (34.3)	16.81 (39.9)
	D-HotJazzClub	12.89 (11.54)	26.75 (30.93)	-3.75 (17.06)	-15.32 (38.15)
	D-SputnikHalle	12.48 (12.2)	32.74 (31.33)	-1 (17.6)	-10.81 (44.42)
E	E-Bauhaus	57.35 (48.7)	45.95 (45.96)	-26.75 (71.28)	-20.25 (62.48)
	E-Hafenkaeserei	43.04 (30.01)	43.36 (40.26)	-37.32 (37.11)	0.14 (59.75)
	E-MrWash	55.12 (40.58)	44.06 (36.71)	8.38 (69.38)	30.56 (49.22)
	E-StJoseph	53.33 (41.28)	43.71 (39.12)	-40.76 (54.28)	-11.62 (58.27)
	F-Franziskus	42.55 (37.13)	32.9 (26.35)	-11.25 (56.14)	8 (42.03)
	F-HBF	24.52 (19.74)	36.76 (44.28)	-10.66 (29.91)	24.83 (52.17)

F		Absolute error		Circular error	
location	landmark	pointing: M (SD)	sketchmap: M (SD)	pointing: M (SD)	sketchmap: M (SD)
	F-Marktkauf	49.86 (33.39)	56.17 (38.86)	18.32 (58.04)	14.87 (67.66)
	F-RoteLolaPub	9 (8.56)	24.29 (37.88)	-3.71 (11.96)	10.86 (43.87)
Exp1	Exp1-Aral	23.88 (28.68)	28.27 (29.73)	-10.16 (36.03)	-4.64 (40.94)
	Exp1-ilDivino	30.83 (29.85)	42.95 (37.33)	16.83 (39.62)	18.74 (53.97)
	Exp1-Mensa	21.36 (20.15)	24.39 (34.69)	-3.67 (29.27)	-5.72 (42.14)
	Exp1-Tormin	33.71 (18.55)	40.38 (27.29)	0.22 (38.73)	14.49 (46.82)

Table 2: Angular deviation of each route with regard to each target. All targets are listed together with the possible route choices and the number of participants who selected that route. This table can be matched with the subsequent map visualisations in order to understand which route ID corresponded to which street on the map.

location	landmark	route ID	angular deviation	how often chosen
A	A-Brillux	1	-157	6
		2	10	7
		3	-37	16
	A-Himmelreich	1	3	21
		2	179	2
		3	122	0
	A-Sparkasse	1	-101	9
		2	72	2
		3	-16	12
	A-Zoo	1	119	11
		2	-64	16
		3	-125	1
B	B-BekenntFarbe	1	-1	28
		2	166	1
		3	87	0
	B-Boeselburg	1	-160	3
		2	13	21
		3	-64	1
	B-Moevenpick	1	115	4
		2	-75	19
		3	-155	1
	B-Wasserturm	1	-80	8
		2	92	3
		3	9	9
	C-Cineplex	1	-111	23
		2	70	2
	C-Ludgeriplatz	1	45	23
		2	-134	2
	C-Suedpark	1	136	5
		2	-42	17

location	landmark	route ID	angular deviation	how often chosen
	C-Verlag	1	-36	18
		2	140	1
D	D-Brasserie	1	10	28
		2	156	0
	D-Gleis22	1	47	27
		2	-179	1
	D-HotJazzClub	1	-61	2
		2	26	26
	D-SputnikHalle	1	-165	1
		2	-30	26
E	E-Bauhaus	1	53	1
		2	83	0
		3	-83	19
	E-Hafenkaeserei	1	115	0
		2	145	0
		3	-18	28
	E-MrWash	1	-107	0
		2	-67	0
		3	-117	16
	E-StJoseph	1	-160	0
		2	-130	0
		3	60	21
F	F-Franziskus	1	-100	18
		2	84	1
		3	175	1
	F-HBF	1	-36	27
		2	147	1
		3	-114	1
	F-Marktkauf	1	113	17
		2	-57	1
		3	39	4
	F-RoteLolaPub	1	4	27
		2	-161	0
		3	-68	1
		1	66	35

location	landmark	route ID	angular deviation	how often chosen
Exp1	Exp1-Ark	2	154	1
		3	-116	21
	Exp1-ilDivino	1	-38	21
		2	45	35
		3	135	3
	Exp1-Mensa	1	144	1
		2	-125	1
		3	-38	57
	Exp1-Tormin	1	-95	8
		2	-9	24
		3	79	25

Map visualisations

Raw data visualized landmark-by-landmark, for each location on a separate page. Lines in circular plots indicate the correct direction to the given landmark.

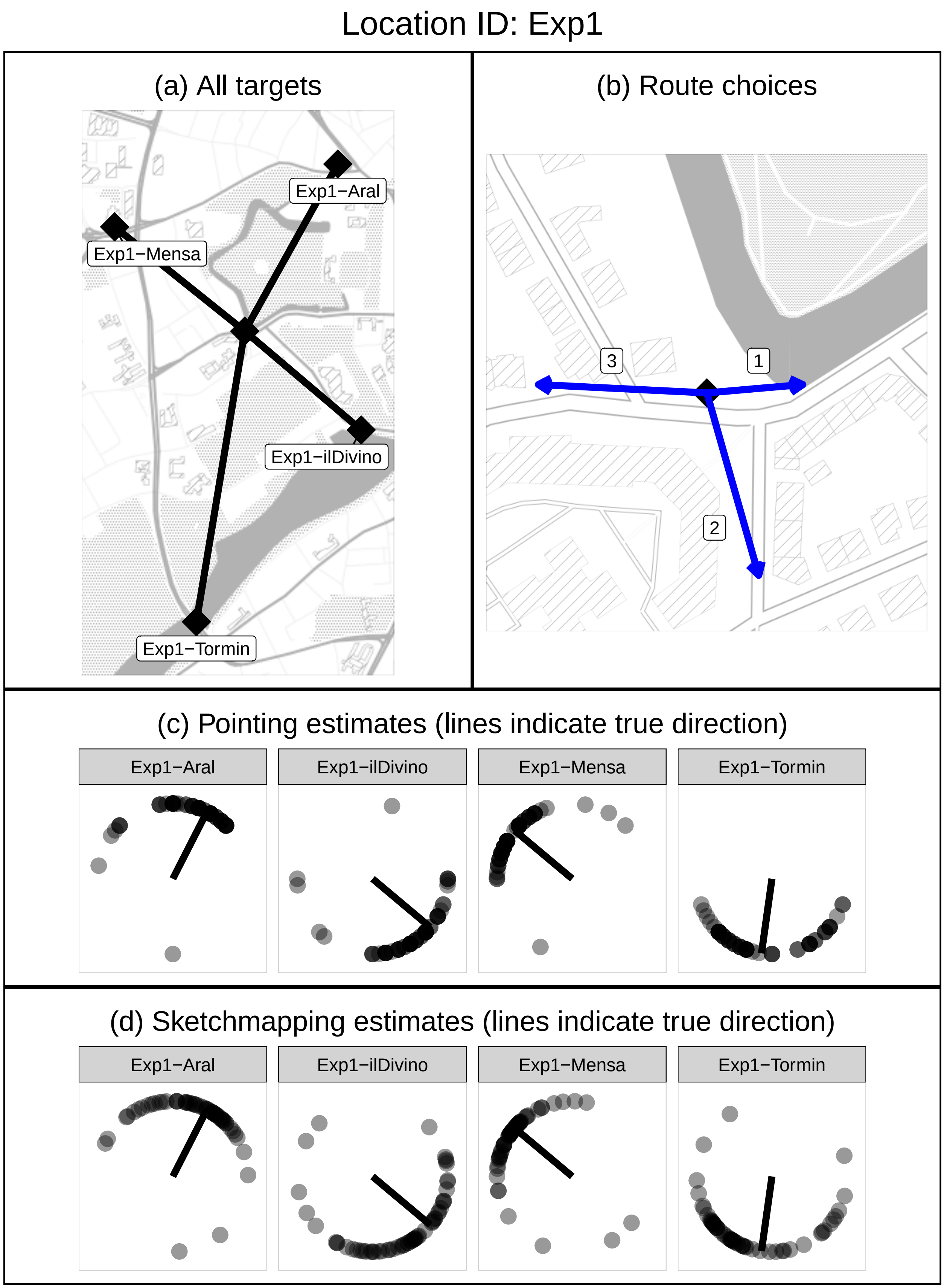

Map tiles by Stamen Design, under CC BY 3.0. Data by OpenStreetMap, under ODbL.

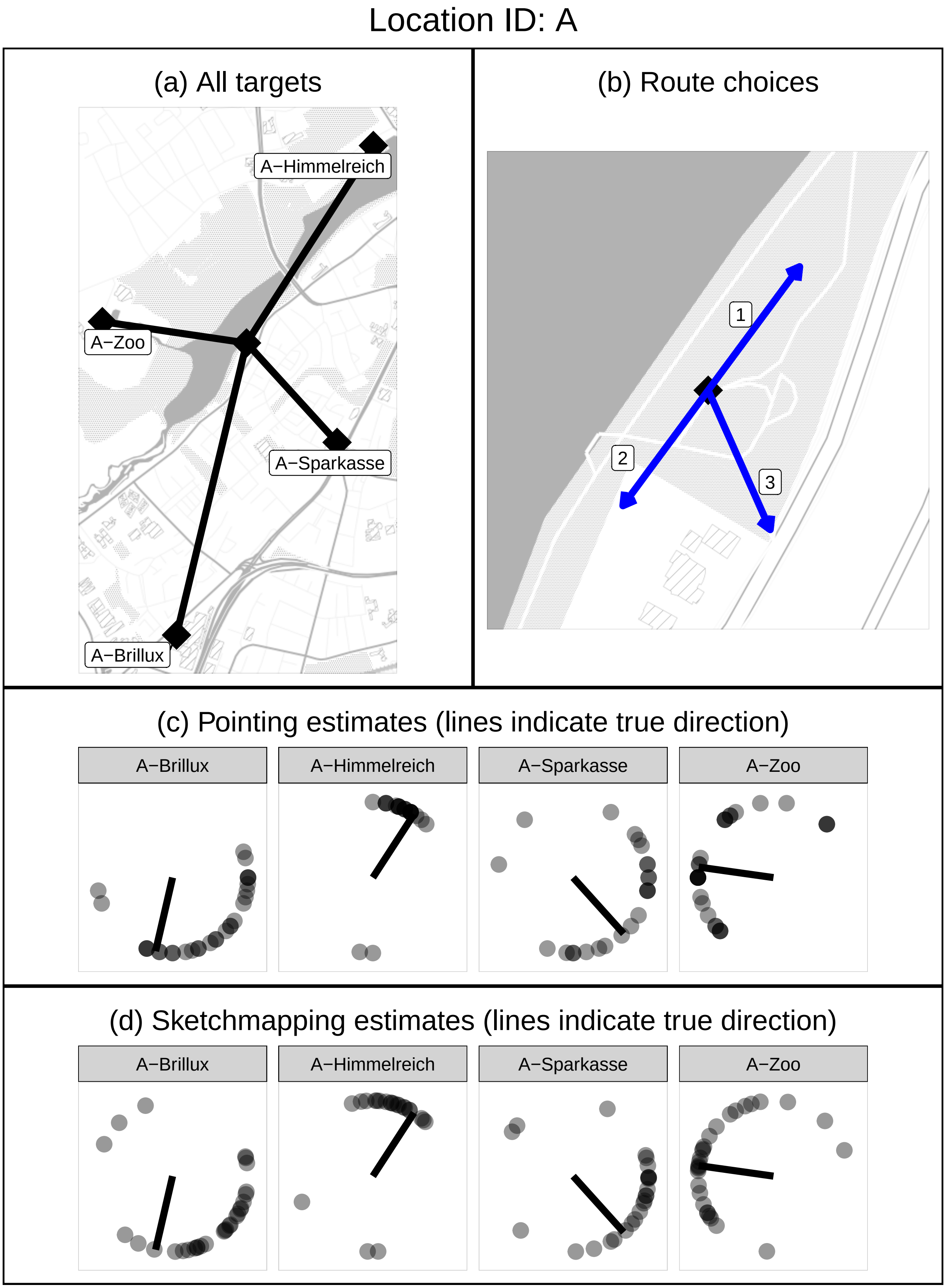

Map tiles by Stamen Design, under CC BY 3.0. Data by OpenStreetMap, under ODbL.

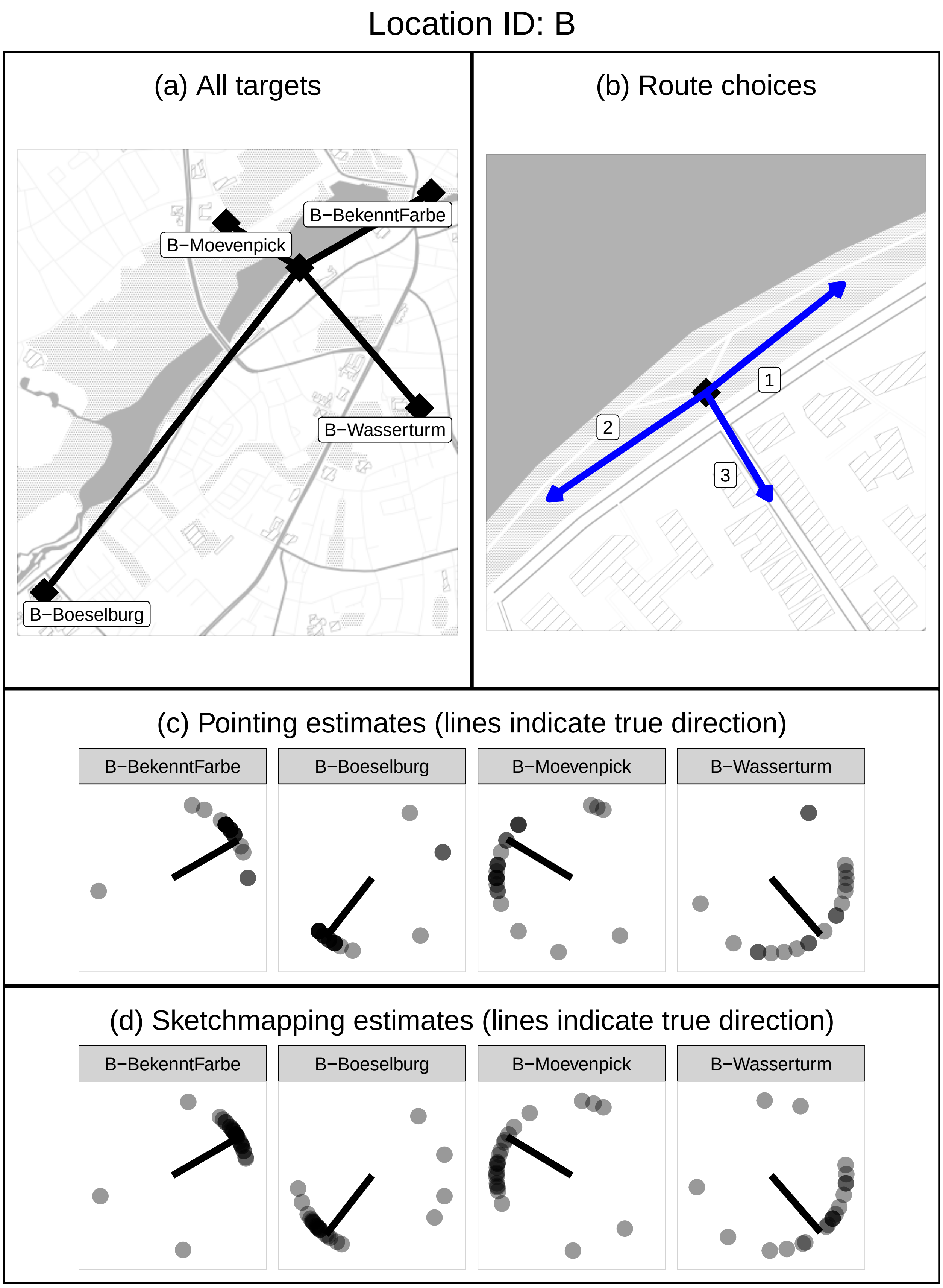

Map tiles by Stamen Design, under CC BY 3.0. Data by OpenStreetMap, under ODbL.

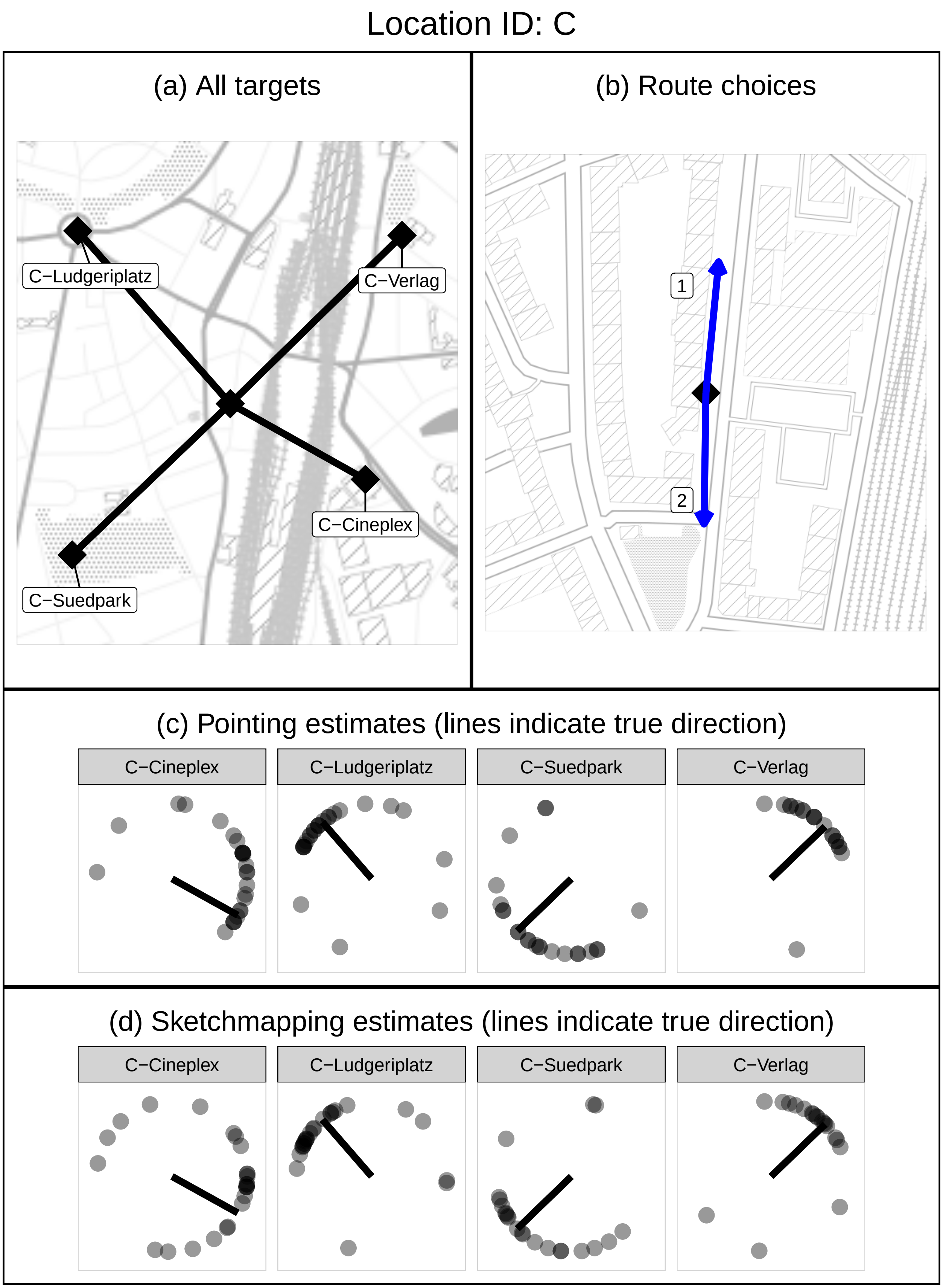

Map tiles by Stamen Design, under CC BY 3.0. Data by OpenStreetMap, under ODbL.

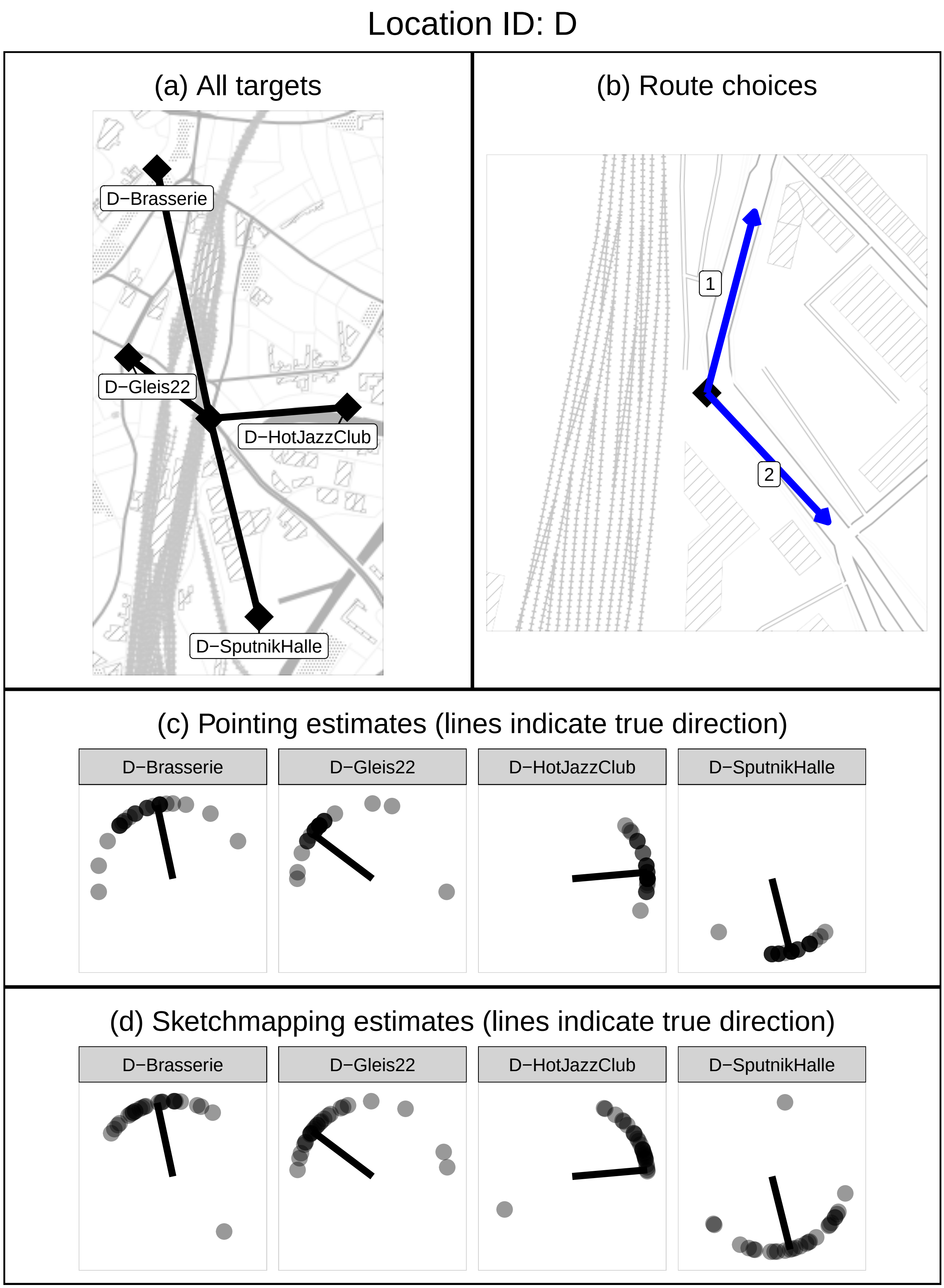

Map tiles by Stamen Design, under CC BY 3.0. Data by OpenStreetMap, under ODbL.

Location ID: E

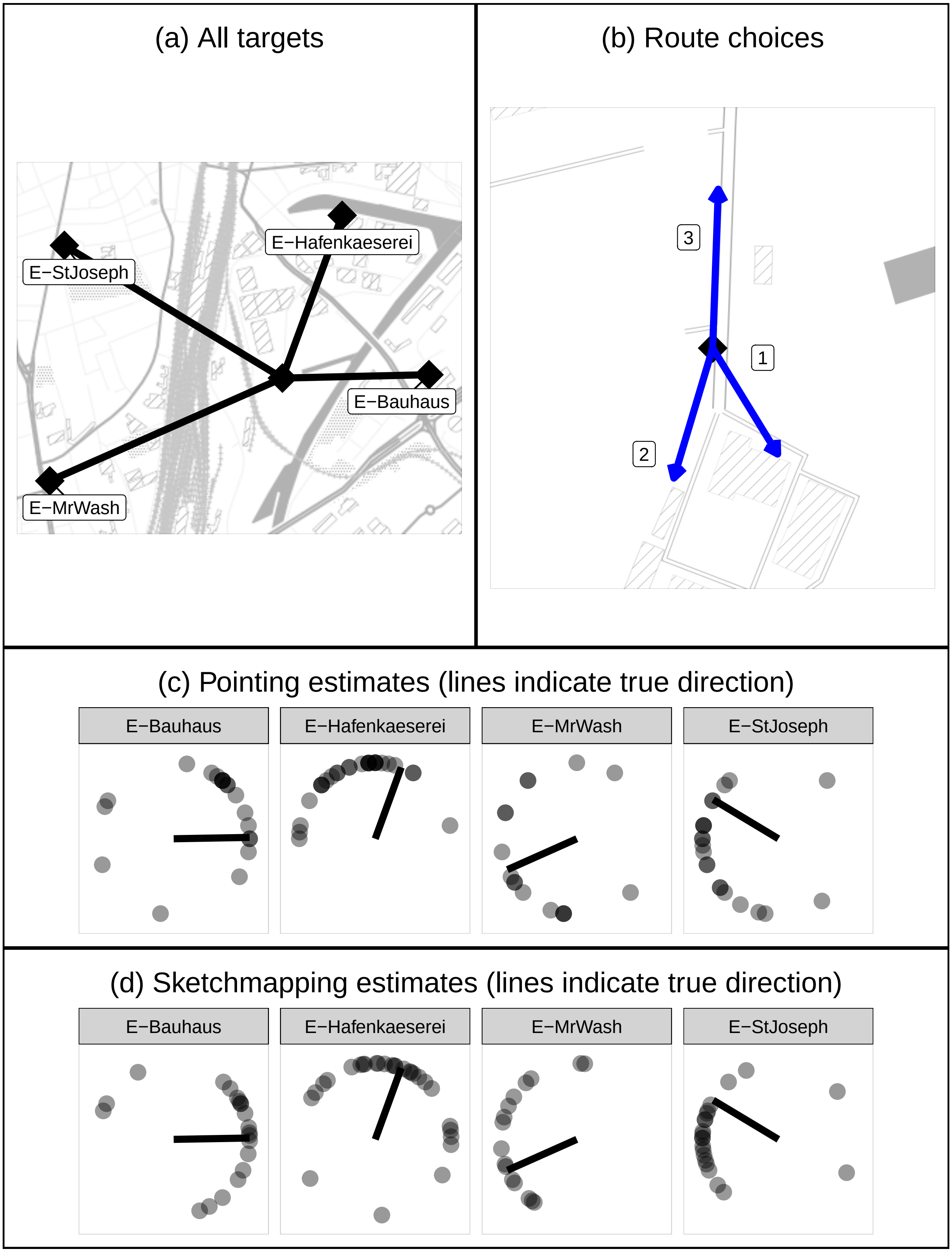

Map tiles by Stamen Design, under CC BY 3.0. Data by OpenStreetMap, under ODbL.

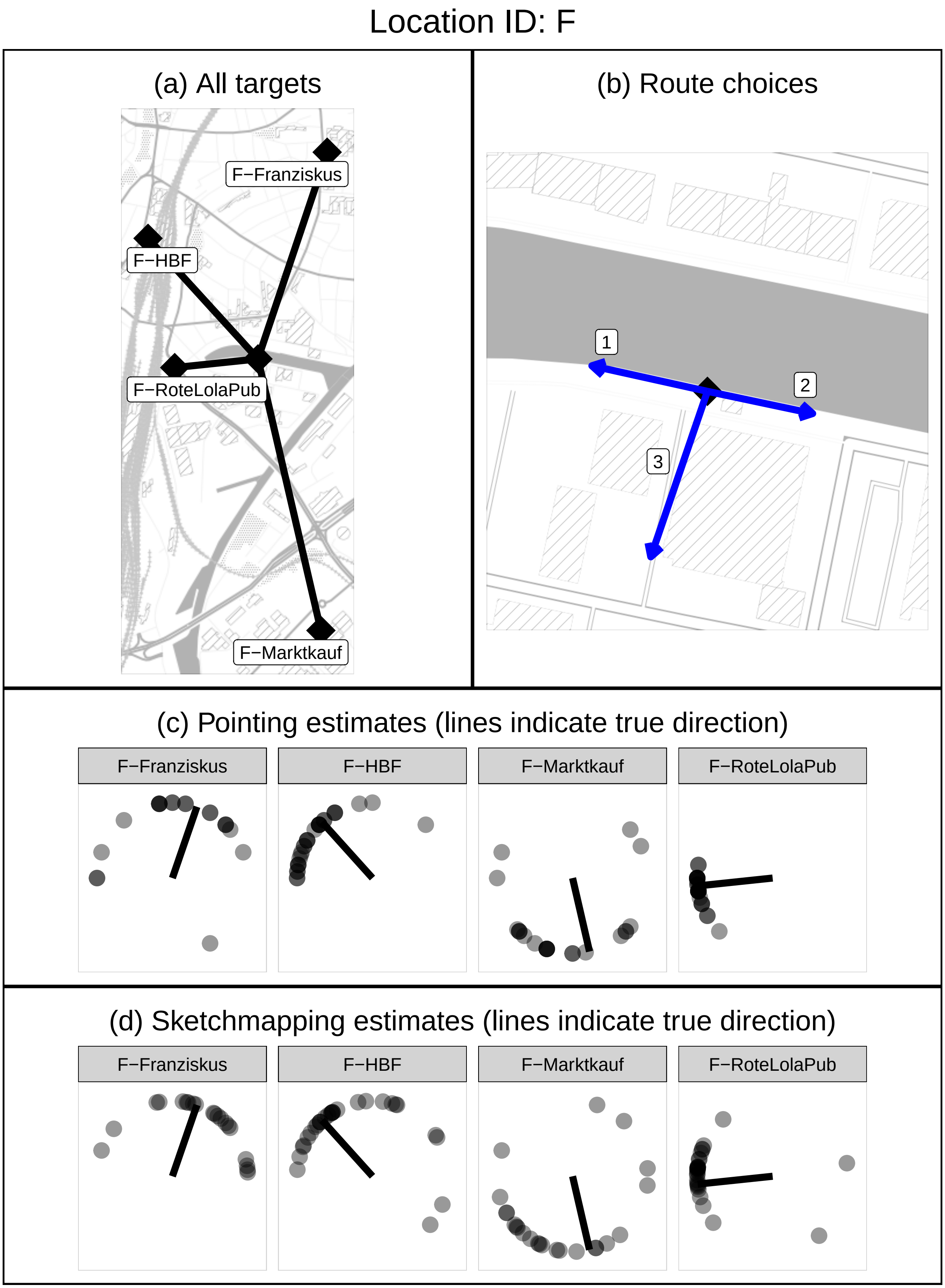

Map tiles by Stamen Design, under CC BY 3.0. Data by OpenStreetMap, under ODbL.
