# Supplementary material for: Route effects in city-based survey knowledge estimates
Source: Cogn Process. 2023 Jan 23;24(2):213–31. doi: 10.1007/s10339-022-01122-0 (PMC10110726; doi:10.1007/s10339-022-01122-0)
Supplement: Supplementary file 2 — (pdf 707 KB) [file 10339_2022_1122_MOESM2_ESM.pdf]

Appendix B

Additional measures for identifying distances to targets across barriers

anonymised

Introduction

The analysis reported in the paper focused on the *angle of the initial route segment* as a measure of route property.

We investigated the possibility that other properties of the route leading to the target have an influence on the pointing/sketchmapping estimates. We computed two additional route measures and applied the same procedure for deriving Bayes Factors for these models, as in the main manuscript.

We perform this comparison only on the *uniform path choice* cases because the alternative measures reported in this appendix are not sensitive to the path chosen by the participant - they provide one value for each location-target pair, regardless of the angular deviation of its initial route segments. Thus, only the *uniform path choice* cases can be compared in a corresponding analysis because they were pre-selected to contain such parts of the dataset, that only have one *angular deviation of the chosen route* value per each location-target pair. We standardised and zero-centred Measure 1 and Measure 2 in order to facilitate faster model fitting.

The results show, that there was no evidence for a significant effect of Measure 1 or 2 on pointing and sketchmapping errors.

Definitions of new measures

Measure 1

We defined Measure 1 as the ratio between the length of a straight line segment $\vec{LM}$ defined by a location point (L) and a landmark point (M) and the length of the line segment $\vec{IF}$ formed by the intersection point (I) of $\vec{LM}$ and the furthest point (F) on the shortest distance walkable route between L and M (Figure 1).

We used the geosphere R package to calculate the geodesic distance between the respective points of our measurement (location to landmark, and route's further point to intersection). We used the Open Route Service Machine (ORSM) with OpenStreetMap data to calculate the shortest route between locations and landmarks. Routes were calculated setting ORSM parameters to use the "walk" profile and exclude ferry routes. Both geosphere and OSRM assume the WGS84 ellipsoid for their calculations.

$$M1 = \frac{\vec{LM}}{\vec{IF}}$$

Measure 2

We defined Measure 2 as the angle ϕ (in degrees) between the line segment $\vec{LM}$ formed by a location point (L) and a landmark point (M) and the line segment $\vec{LF}$ formed by L and the furthest point (F) of the shortest distance walkable route between the location and the landmark (Figure 2).

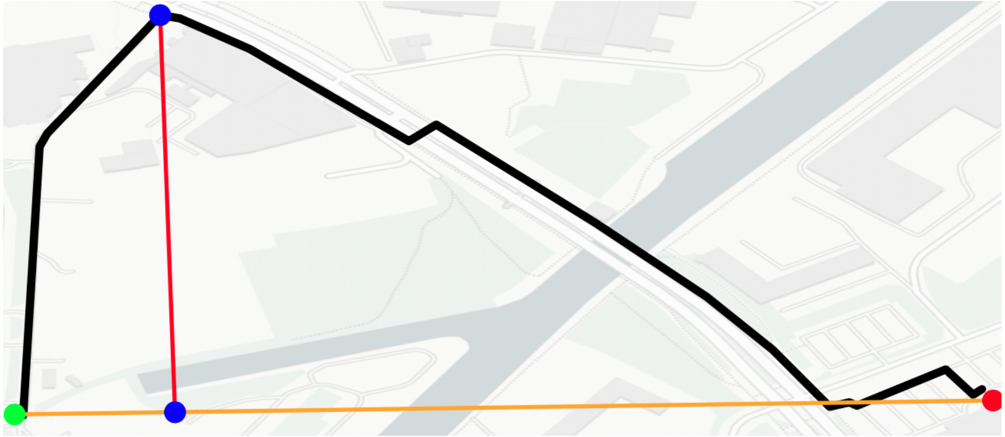

Figure 1: Deriving Measure 1. Black line: Shortest distance walkable route between location E (green point) and landmark Bauhaus (red point). Orange line: LM line segment between the location point (here: E) and the landmark point (here: Bauhaus). Red line: IF line segment defined by the point on the route that is the furthest with regard to the LM segment and the intersection point (both points marked in blue). Measure 1 is the ratio of lengths of both lines.

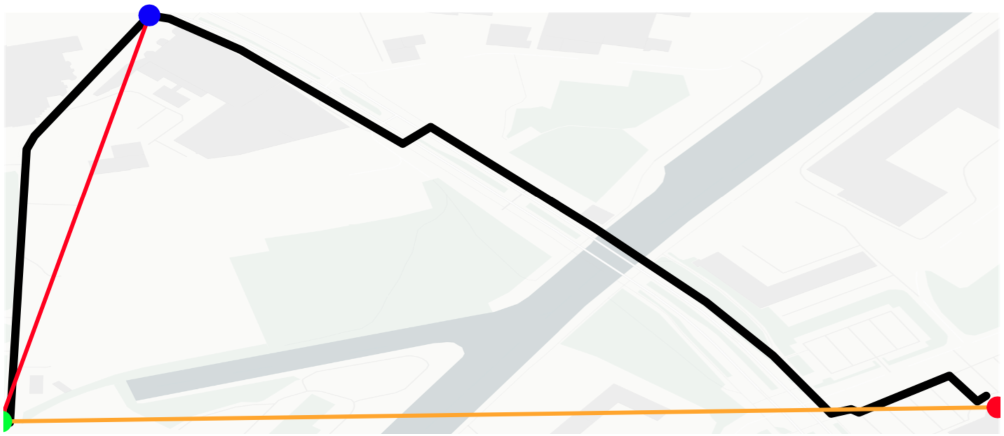

Figure 2: Deriving Measure 2. Black line: Shortest distance walkable route between location E (green point) and landmark Bauhaus (red point). Orange line: LM line segment between the location point (here: E) and the landmark point (here: Bauhaus). Red line: LF line segment defined by the point on the route (blue point) that is the furthest with regard to the LM segment and the location point E (green point). Measure 2 is the angle between the two lines.

Table 1: Summary statistics of Measures 1 and 2.

landmark	Measure 1	Measure 2
A-Himmelreich	0.18	14.42
B-BekenntFarbe	0.09	5.84
B-Boeselburg	0.03	2.02
B-Moevenpick	1.20	80.41
C-Cineplex	0.56	47.96
C-Ludgeriplatz	0.26	53.22
C-Suedpark	0.20	14.53
C-Verlag	0.38	29.84
D-Brasserie	0.32	23.72
D-Gleis22	0.29	57.67
D-HotJazzClub	0.18	59.43
D-SputnikHalle	0.24	26.25
E-Bauhaus	0.66	75.97
E-Hafenkaeserei	0.24	17.42
E-MrWash	1.19	44.31
E-StJoseph	0.61	47.73
F-Franziskus	0.27	107.77
F-HBF	0.26	29.21
F-Marktkauf	0.17	43.58
F-RoteLolaPub	0.14	10.79

We used geosphere R package to calculate the bearing (direction; azimuth) between the respective points of our measurement (location to landmark, and location to route's furthest point). We used the Open Route Service Machine (ORSM) with OpenStreetMap data to calculate the shortest route between locations and landmarks. Routes were calculated setting ORSM parameters to use the "walk" profile and exclude ferry routes. Both geosphere and OSRM assume the WGS84 ellipsoid for their calculations.

Results

Table 1 contains the summary of all values calculated according to the procedures described above.

Pointing: Measure 1

There was very strong evidence (BF = 1/65.27) against the hypothesis stating a circular correlation between Measure 1 and the pointing error.

Pointing: Measure 2

There was very strong evidence (BF = 1/60.98) against the hypothesis stating a circular correlation between Measure 2 and the pointing error.

Sketchmapping: Measure 1

There was extreme evidence (BF = 1/166.14) against the hypothesis stating a circular correlation between Measure 1 and the sketchmapping error.

Sketchmapping: Measure 2

There was extreme evidence (BF = 1/102.41) against the hypothesis stating a circular correlation between Measure 2 and the sketchmapping error.
